# Metabolic environment-driven remodeling of mitochondrial ribosomes regulates translation and biogenesis

**DOI:** 10.1016/j.molcel.2025.10.012

**Published:** 2025-11-06

**Authors:** Fan Zheng, Zanlin Yu, Junming Zhuang, Lingraj Vannur, William Nickols, YongQiang Wang, Liying Li, Sho Joseph Ozaki Tan, Seungmin Yoo, Yifan Cheng, Chuankai Zhou

**Affiliations:** 1 Buck Institute for Research on Aging, 8001 Redwood Blvd., Novato, CA 94945, USA; 2 Department of Biochemistry and Biophysics, University of California San Francisco, 600 16th Street, San Francisco, CA 94158, USA; 3 Howard Hughes Medical Institute, University of California San Francisco, San Francisco, CA 94158, USA; 4 Present address: CryoEM Core Facility, National Institute of Neurological Disorders and Stroke, National Institutes of Health, Bethesda, MD 20892, USA; 5 Harvard T.H. Chan School of Public Health, Harvard University, 677 Huntington Ave, Boston, MA 02115, USA; 6 These authors contributed equally; 7 Lead contact

## Abstract

Cytosolic translation activity is fine-tuned by environmental conditions primarily through signaling pathways that target translation initiation factors. Although mitochondria possess their own translation machinery, they lack an autonomous signaling network analogous to their cytosolic counterpart for regulating translation activity. Consequently, our understanding of how mitochondrial translation activity is adjusted under different metabolic environments remains very limited. Here, we report a noncanonical mechanism for regulating mitochondrial translation activity via metabolism-dependent changes in the mitochondrial ribosome (mitoribosome) in *S. cerevisiae*. These changes arise from a metabolism-modulated mitoribosome assembly pathway that regulates the composition and conformation of the mitoribosome, thereby adjusting its translation activity to meet metabolic demands. Moreover, the translation activity of the mitoribosome feeds back to regulate the biogenesis of nuclear-encoded mitochondrial proteins, influencing mitochondrial functions and aging. Such a ribosomal remodeling-based “gear-switching” mechanism represents an orthogonal mode of translation regulation, compensating for the absence of a translation-modulating signaling network within mitochondria.

## In brief

Zheng et al. demonstrate that mitochondria remodel the structure and composition of mitochondrial ribosomes in response to metabolic state. This structural “gear-switching” mechanism fine-tunes mitochondrial translation and biogenesis, compensating for the absence of dedicated signaling pathways within mitochondria.

## Graphical Abstract

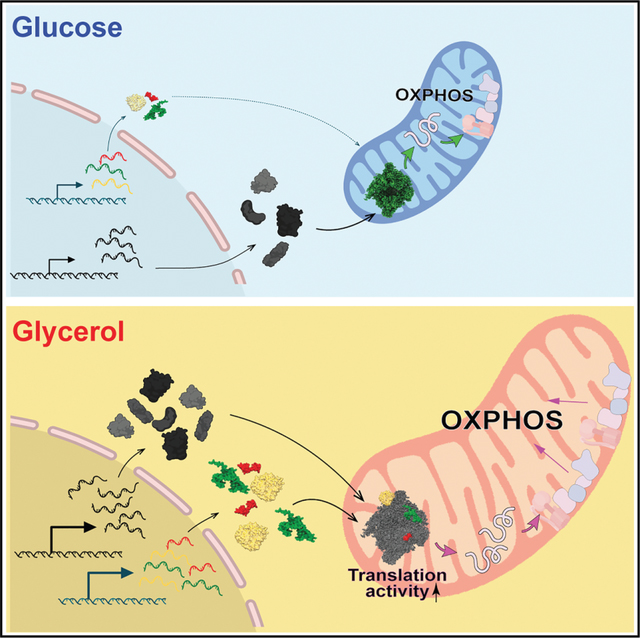

## INTRODUCTION

Organisms demonstrate remarkable adaptability to environmental stimuli by modulating gene expression to thrive under changing conditions. For instance, cells fine-tune mitochondrial biogenesis and function to adjust energy production pathways based on available carbon sources. Cells rely on ATP generated by the mitochondrial oxidative phosphorylation system (OXPHOS), which includes the electron transport chain (ETC) and F-ATPase, to thrive on non-fermentable carbon sources such as glycerol. Conversely, when fermentable sugars like glucose are available, many cells—including cancer cells—predominantly utilize glycolysis for rapid ATP production, a metabolic shift known as the Crabtree effect or Warburg effect.^1,2^ Under glucose-rich conditions, OXPHOS complex biogenesis is suppressed, and its contribution to the overall ATP supply is significantly reduced, with the majority being produced through glycolysis.^1^ By contrast, cells enhance mitochondrial biogenesis and OXPHOS complex production under non-fermentable conditions.^3^

Unlike the robust and extensive cytosolic signaling pathways that regulate nuclear gene expression, mitochondria lack an autonomous signaling network for fine-tuning their own gene expression. For example, while cytosolic translation is modulated by environmental conditions through signaling pathways that target translation initiation factors (e.g., eIF2α and 4E-BP),^4^ translation of mitochondrial DNA (mtDNA)-encoded OXPHOS proteins within mitochondria is thought to be primarily controlled by nuclear-encoded OXPHOS subunits produced by cytosolic translation, thereby maintaining balanced production of OXPHOS subunits from two genomes.^5,6^ However, recent findings indicate that nuclear-encoded OXPHOS subunits are substantially over-synthesized relative to mtDNA-encoded subunits,^7^ suggesting that the conventional model, where mitochondrial translation is regulated by the availability of nuclear-encoded OXPHOS subunits, may be incomplete. Consequently, additional regulatory mechanisms of mitochondrial translation likely exist that operate independently of—or in parallel with—the availability of cytosol-translated OXPHOS subunits. Indeed, evidence for mitochondria-derived regulation has emerged, as the overexpression of mitochondrial transcription factors can somehow enhance mitochondrial translation.^8–13^ Compared with the well-characterized regulation of cytosolic translation, our understanding of how mitochondrial translation activity is adjusted under different metabolic environments—ensuring adequate production of mtDNA-encoded proteins to fulfill metabolic demands—remains very limited.

Mitochondrial translation is performed by the mitochondrial ribosome (mitoribosome), which is assembled from mtDNA-encoded rRNA and nuclear-encoded mitoribosome proteins (MRPs).^14–17^ While mitochondrial biogenesis and ultrastructure are known to adapt closely to metabolic demands and environmental signals, the prevailing view assumes that the mitoribosome remains the same in both structure and function. This perspective describes the mitoribosome as merely a protein synthesis machine, with its translation being activated by the availability of nuclear-encoded OXPHOS subunits.^5^ Here, we use the mitoribosome from budding yeast *Saccharomyces cerevisiae* as a model to demonstrate that growth conditions and metabolic contexts change the composition and structure of rRNAs and MRPs to adjust the activity of mitoribosomes. Notably, changing mitochondrial translation activity feeds back to control the biogenesis of nuclear-encoded mitochondrial proteins. Thus, compared with cytosolic ribosomes, whose translation activity is regulated by signaling cascades, mitochondria have evolved unique mechanisms to modulate mitoribosome translation activity to fine-tune both mtDNA and nuclear gene expression.

## RESULTS

### Determine the context-dependent structures of mitoribosomes

Given that the mitochondrial proteome, ultrastructure, and metabolism are profoundly restructured by the available carbon sources in the environment, we investigated whether and how different carbon sources, such as fermentable (YP-dextrose [YPD]) and non-fermentable (YP-glycerol [YPG]) carbon sources, impact mitoribosomes structurally and functionally. Published yeast mitoribosome structures were determined from cells cultured in YPG.^15^ We thus started to address this question by determining the structure of mitoribosomes from yeast grown in YPD and compared it with the published mitoribosome structure from cells grown in YPG.

**Figure 1.**
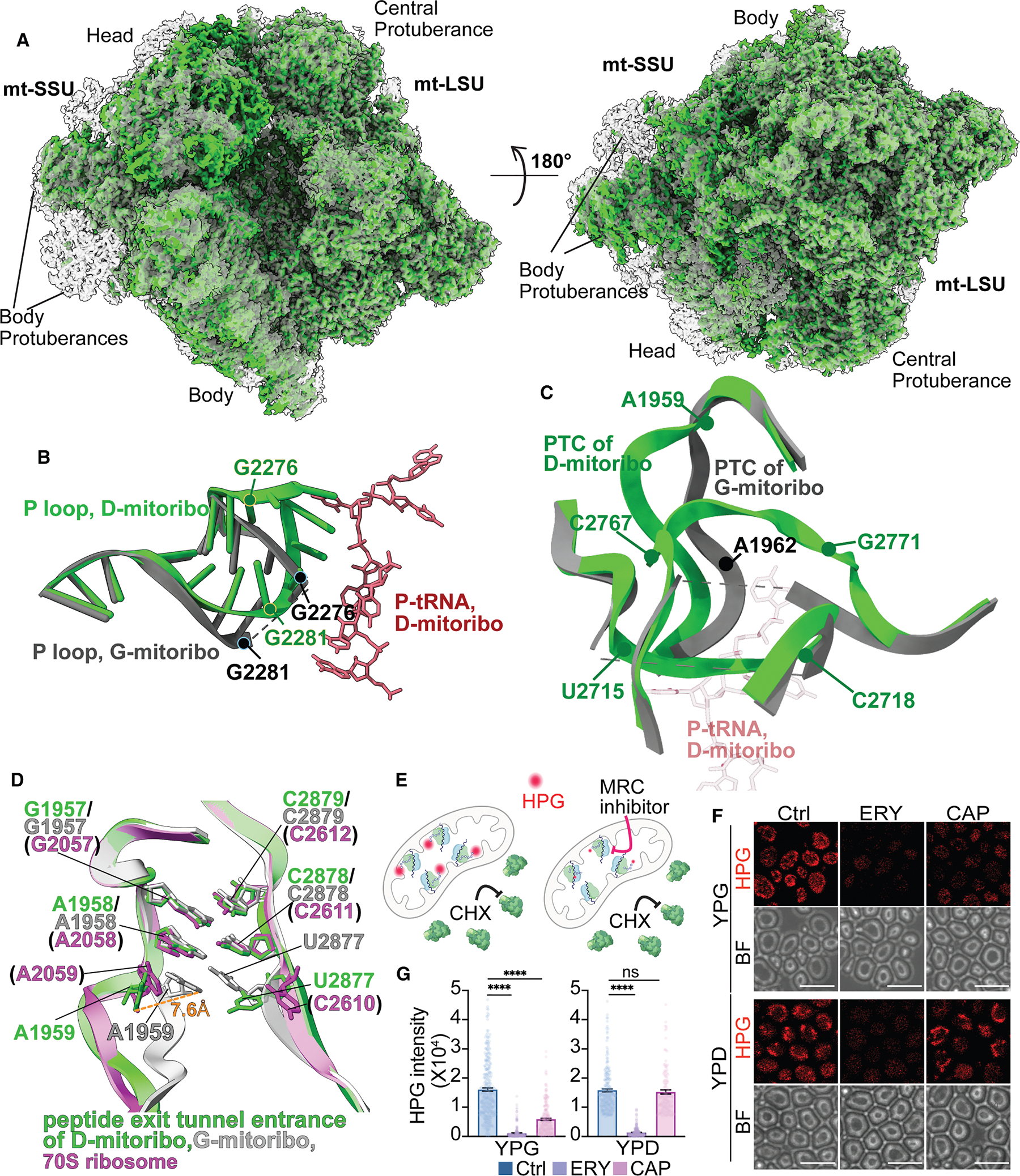
The structural difference of 21S rRNA between D- and G-mitoribosome (A) Overall map comparison of D-mitoribosome (green) and G-mitoribosome (gray, from EMDB: EMD-3551). (B) Comparison of P loop from D-mitoribosome (green) and G-mitoribosome (gray, from PDB: 5MRC, same for other figures). The nucleotides from 2277 to 2280 in the P loop are missing from G-mitoribosome. See Figure S1F for density maps. Mitoribo, mitoribosome (same for other figures). (C) Structural difference of the PTC region between D-mitoribosome (green) and G-mitoribosome (gray). See Figure S1G and Video S1 for density maps. (D) Comparison of peptide exit tunnel entrance in D-mitoribosome (green) and G-mitoribosome (gray) and bacterial 70S ribosome (pink, from PDB: 7K00). (E) Schematic illustration of Mito-FUNCAT assay in yeast. Cycloheximide (CHX) is added to inhibit the cytosolic ribosome translation, allowing the incorporation of HPG (an analog of methionine) into the newly synthesized proteins selectively within mitochondria. Click reactions between the alkyne moiety of HPG and azide of Alexa Fluor 594 reveal the nascent peptide translated by mitoribosomes inside the mitochondria. (F and G) Representative Mito-FUNCAT images (F) and quantification (G) of yeast cells treated with different drugs. Images are representative of at least three independent experiments. Shown in (G) are mean and SEM (cell numbers for each strain refer to Table S2). Ctrl, control cells exposed to CHX but no mitoribosome inhibitors. Scale bar: 10 μm. Images were collected with different settings for YPD and YPG cells due to different brightness of signal. Within the YPD or YPG group, the imaging setting is the same for control and inhibitor-treated cells.

We purified mitoribosomes from yeast grown in YPD (D-mitoribosome) by affinity pull-down of the endogenously tagged large subunit protein bL17m and subjected them to structural studies using single-particle cryogenic electron microscopy (cryo-EM). After multiple rounds of 3D classification, we successfully isolated intact 74S mitoribosome particles and obtained two high-resolution reconstructions with different conformations (~3.1 to 3.3 Å, classes I and II; Figure S1A). Between them, the large subunit (mtLSU) remains almost identical, while the orientation of the small subunit (mtSSU) relative to the mtLSU differs by approximately 5.2° (Figures S1B–S1D). This difference in the relative orientation of the mtSSU corresponds to two distinct translation states: class I, containing a P-site tRNA and mRNA, and class II, containing an E-site tRNA and mRNA (Figure S1B). Given the high similarity of classes I and II, we focused on the mitoribosome class I with a P-site tRNA in this study. The presence of mRNA and tRNA indicates that these D-mitoribosomes are translationally active (Figures S1B and S1E). The resolution and map quality of both reconstructions are sufficient for accurate model building and refinement, as well as for accurate comparisons with previously determined mitoribosome structures from YPG (G-mitoribosome) (Figure 1A).^15^ We built the atomic models of D-mitoribosomes using G-mitoribosome as the initial input and refined them against each D-mitoribosome map. The complete models of D-mitoribosomes include two rRNA molecules (21S rRNA in the mtLSU and 15S rRNA in the mtSSU), 72 MRPs, and tRNAs (Table S1).

### D-Mitoribosome differs from G-mitoribosome in PTC, P loop, and peptide exit tunnel

Noticeable differences were observed in several critical regions of 21S rRNAs in the mtLSU between D- and G-mitoribosomes, including the P loop (nucleotides 2,274–2,282) and peptidyl transferase center (PTC) (nucleotides 1,958–1,964, 2,713–2,720, and 2,765–2,773) (Figures 1B and 1C). Part of these loops (nucleotides 2,277–2,280, 2,715–2,718, and 2,767–2,771) are not modeled in G-mitoribosome due to the lack of density in the map^15^ (Figures S1F–S1H; Video S1). The nucleotides 1,958–1,964 of PTC, which were captured in both D- and G-mitoribosomes, fold into distinct structures in D- and G-mitoribosomes (Figure 1C). While the PTC nucleotides 2,715–2,718 and 2,767–2,771 were not captured in the G-mitoribosome, which prevents direct comparison, the surrounding nucleotides of these two loops show structural differences between G- and D-mitoribosomes, indicating that these two PTC loops also have distinct structures between cells cultured in different carbon sources (Figure 1C; Video S1).

In addition to the difference in the P loop and PTC, D-mitoribosome also differs significantly from G-mitoribosome at the entrance to the peptide exit tunnel (Figures 1D and S1I). In the D-mitoribosome, nucleotide A1959 shifts 7.6 Å from its position in the G-mitoribosome, widening the gap between the opposing strands of 21S rRNA surrounding the entrance (Figure 1D). Remarkably, the D-mitoribosome entrance configuration closely resembles that of the bacterial 70S ribosome—the ancestral form of the mitoribosome—and the human mitoribosome (Figures 1D and S1J), suggesting an evolutionary conservation of the peptide exit tunnel entrance across different species and ribosomal types. However, the structural changes in the P loop, PTC, and peptide exit tunnel entrance also reveal an intriguing aspect of mitoribosome evolution: while maintaining the basic conserved structure, the mitoribosome has developed additional flexibility, allowing the same rRNA sequence to adopt different conformations under varying metabolic contexts.

### rRNA structural differences in PTC affect mitoribosome’s sensitivity to rRNA-interacting antibiotics

To assess whether the structural differences in the PTC and peptide exit tunnel are functionally relevant and independent of the translation statuses of the mitoribosome structures, we examined the sensitivity of mitoribosomes to two well-known antibiotics, chloramphenicol (CAP) and erythromycin (ERY). These antibiotics are known to interact with the PTC pocket and peptide exit tunnel, respectively, inhibiting translation in both bacterial and mitoribosomes.^18–23^ In bacterial 70S ribosome, CAP binds to the PTC via hydrogen bonds with A2062 (A1962 in yeast mitoribosome) and G2505 (G2771 in yeast mitoribosome).^24^ Since mutations of A2062 and G2505 in bacteria are known to reduce CAP’s inhibitory efficacy on ribosomes, the conformation difference in these nucleotides between G- and D-mitoribosomes predicts that CAP likely has different effects on them (Figure 1C).^24–27^ By contrast, the A2058 (A1958 in yeast mitoribosome), the key nucleotide for the sensitivity and resistance to ERY,^24^ remains unchanged in D-mitoribosome compared with G-mitoribosome (Figure 1D), predicting a comparable sensitivity of D- and G-mitoribosomes to ERY.

To test these predictions, we used a modified mitochondria fluorescent noncanonical amino acid tagging (Mito-FUNCAT) protocol to monitor the translation activity of mitoribosomes in yeast cells in the presence or absence of these antibiotics.^28,29^ This method employs L-homopropargylglycine (HPG), a methionine analog, to label newly synthesized polypeptides, specifically reporting mitochondrial translation activity in cells treated with cycloheximide (CHX) to block cytosolic translation (Figure 1E). Indeed, while ERY can efficiently block mitochondrial translation in cells cultured with both glycerol and glucose, CAP selectively suppresses mitochondrial translation in glycerol-grown cells and has only a limited inhibitory effect on mitochondrial translation in YPD-cultured cells (Figures 1F and 1G). CAP’s inability to inhibit growth in glucose medium was previously attributed to cellular insensitivity to mitochondrial defects in the presence of glucose (Figure S1K).^30^ Our results, however, indicate a different explanation: CAP lacks inhibitory activity on D-mitoribosome, the mitoribosome formed under glucose growth conditions.

We used Bi-Genomic Mitochondrial-Split-GFP (BiG Mito-Split-GFP)^31^ to validate this finding. In this system, the first ten β-strands of GFP (GFP_1–10_) are encoded by mtDNA and translated by mitoribosomes inside the mitochondria. The mitochondria-targeted, mCherry-tagged eleventh β-strand of GFP (GFP_11_) is overexpressed (OE) from the nuclear genome. The reconstitution of GFP_1–10_ with GFP_11_ produces fluorescent GFP, thus reporting the abundance of GFP_1–10_ and mitochondrial translation activity (Figure S1L). ERY treatment led to the depletion of the GFP signal in cells cultured in glucose medium (Figures S1M and S1N). As ERY did not affect yeast growth in the glucose medium, the loss of GFP signal is likely due to the inhibition of new GFP_1–10_ synthesis while existing GFPs are diluted and degraded upon the growth and segregation of the mitochondrial network during cell division (Figures S1K and S1O).^32^ This was not caused by the defects in synthesis or import of mCherry-GFP_11_ (Figure S1M), and the abundance of GFP_1–10_ was validated directly with antibody against GFP_1–10_ (Figures S1P and S1Q). By contrast, cells treated with CAP showed limited reduction of GFP signal even though the cells divided twice during the experiments, during which the mitochondrial mass increased four times, which diluted the existing GFP four times (Figures S1M–S1Q).^32^ Thus, compared with ERY-treated cells, the persistent GFP signal in CAP-treated cells indicates a constant synthesis of new GFP_1–10_ throughout cell cycles, consistent with the observation from Mito-FUNCAT that CAP has a limited inhibitory effect on the mitoribosome in cells cultured in glucose medium. In glycerol-grown cells, neither antibiotic reduced GFP, likely because growth inhibition prevented the dilution of existing GFPs (Figures S1K and S1M–S1Q). These findings suggest that the effectiveness of drugs may vary with the environment due to the structural changes of the target.

As there is no available reference structure of a mitoribosome bound to antibiotics, we used bacterial ribosome structures bound to CAP and ERY as references to evaluate how nucleotide conformational changes may influence mitoribosome sensitivity to these drugs. As expected, comparison of the ERY-binding site A1958 (corresponding to A2058 in the 70S ribosome) revealed that this nucleotide adopts a similar conformation in both D- and G-mitoribosomes, closely resembling that in the bacterial ribosome (Figure S1S). This observation is consistent with the comparable inhibitory effect of ERY observed in both conditions. By contrast, we observed that A1962 adopts different conformations in the D- and G-mitoribosomes, and in both cases these conformations differ from that of A2062 in the bacterial ribosome bound to CAP (Figure S1T). This is likely due to the fact that A2062 is a highly flexible nucleotide that adopts different conformations depending on the presence of antibiotics, nascent peptides, or other structural features.^33–37^ For example, A2062 adopts a distinct conformation in antibiotic-free bacterial ribosomes (Figure S1T). This prevents direct comparison between antibiotic-free mitoribosomes and the bacterial ribosome bound to antibiotics. We were unable to evaluate the second CAP-binding site, G2505 (G2771 in the mitoribosome), due to the absence of G2771 in the G-mitoribosome model. Nevertheless, the distinct conformation of A1962 and structural differences surrounding G2771 between D- and G-mitoribosomes suggest that CAP binding at the PTC region likely differs between the two mitoribosome types. This structural difference is consistent with the differential inhibitory effect of CAP on translation activity in D- and G-mitoribosomes (Figures 1F and S1M–S1O).

### The compositional and conformational difference in MRPs

**Figure 2.**
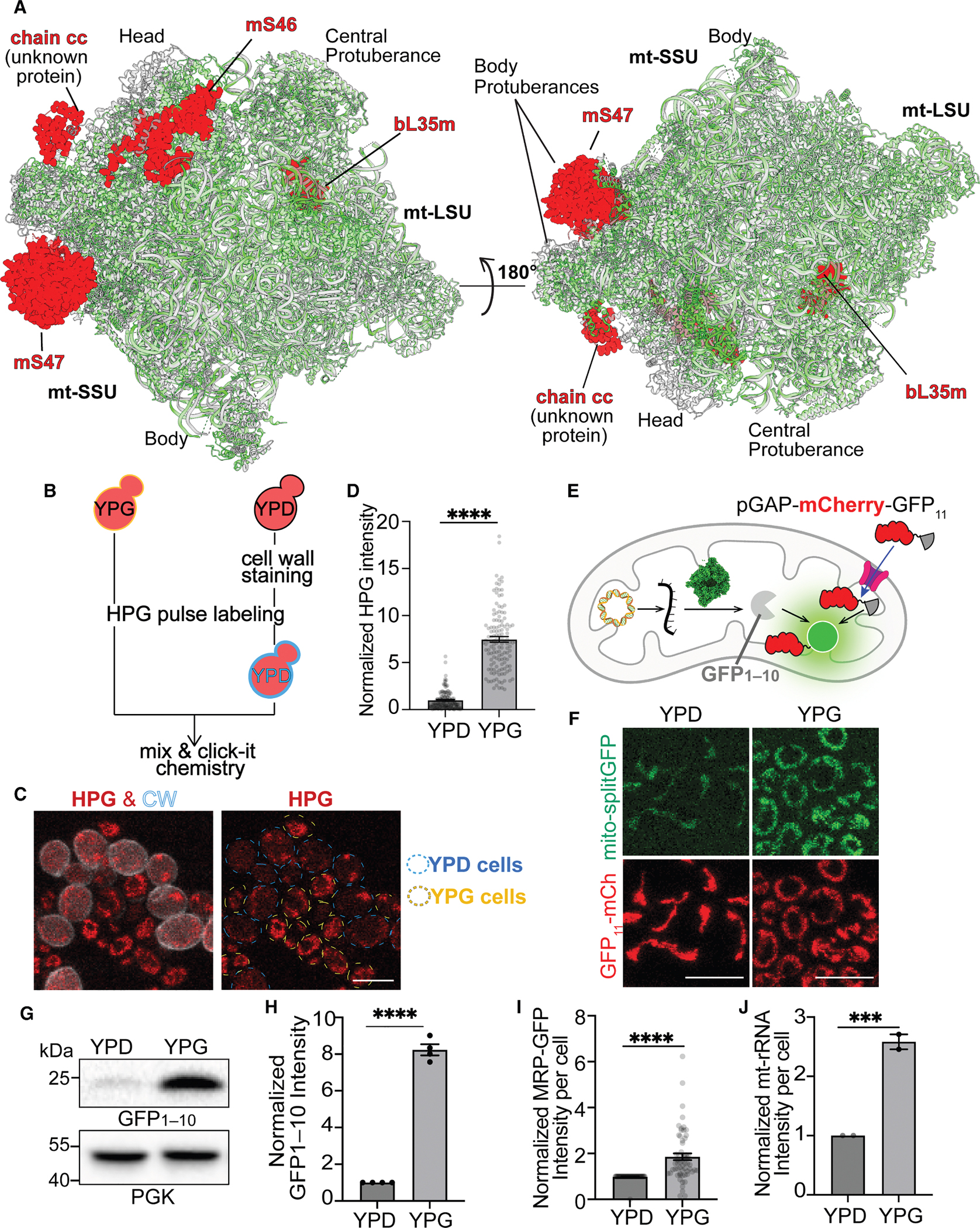
The structural and activity difference between D- and G-mitoribosome (A) Comparison of overall structure of D-mitoribosome (green) and G-mitoribosome (gray). The MRPs not found in D-mitoribosome but existing in G-mitoribosome are marked in red. Additionally, chain cc represents a density only observed in the G-mitoribosome with an unknown identity.^15^ (B) Workflow illustration for measuring the translation activity for mixed cells grown in either YPD or YPG. (C and D) Representative images and quantification of HPG signal in yeast cells grown in glucose and glycerol conditions. Cells grown in YPD were stained with Calcofluor White (CW) to differentiate them from those grown in YPG. Individual channels of CW and HPG are merged and displayed as a composite. Scale bar: 5 μm. (E and F) Schematic illustration and representative images of the mtDNA-encoded GFP_1–10_ (BiG Mito-Split-GFP signal) in cells cultured in YPD and YPG. The abundance of GFP_1–10_ was also verified by western blot (Figures 2G and 2H). Scale bar: 10 μm. (G and H) Western blot and quantification of the abundance of GFP_1–10_ from the samples in (F). (I) Normalized intensity of 54 different MRP-GFPs in yeast cells grown in YPD and YPG. (J) Normalized abundance of mt-rRNA quantified by RT-PCR from the cells grown in YPD and YPG. Bar graphs are mean and SEM (cell numbers are in Table S2).

D-mitoribosomes also differ from G-mitoribosomes by the absence of mS46 and mS47 in the mtSSU and bL35m in the mtLSU (Figures 2A, S2A, and S2B). mS46 is partially buried inside G-mitoribosome by uS10m and h33. Additionally, bL35m is deeply embedded within G-mitoribosome mtLSU by the H31, H38, and H86 of 21S rRNA and numerous associated MRPs. The high resolution of uS10m, h33, H31, H38, H86, and the surrounding rRNA-associated MRPs in the cryo-EM structure of the G- and D-mitoribosome suggests that these elements form stable structures within the mitoribosomes,^15^ making it unlikely for mS46 or bL35m to be released after the assembly of the mitoribosomes. This inherent stability indicates that the probability of nonspecifically losing even a single MRP during sample preparation is low. Consequently, the simultaneous absence of multiple MRPs in the cryo-EM structure of the D-mitoribosome is therefore statistically improbable, indicating that their exclusion reflects a deliberate and biologically relevant compositional difference. It is worth noting that, in addition to the main population of D-mitoribosomes lacking mS47, mS46, and bL35m, we identified a minor (11.5%) population of mitoribosome particles containing mS47 in glucose culture using focused classification (Figure S2C). We did not detect a subpopulation of mitoribosomes containing mS46 and bL35m. What are the biological implications of these mitoribosome compositional differences? We hypothesize that the absence of mS46, mS47, and bL35m in D-mitoribosomes impacts their translation activity, as mitochondria synthesize substantially more OXPHOS subunits in glycerol than in glucose medium due to increased biogenesis and metabolic demands.^3^ To test this hypothesis, we next investigate whether D- and G-mitoribosomes exhibit different translation activities.

### D-Mitoribosome differs from G-mitoribosome in translation activity

To quantitatively compare translation activities between different mitoribosomes, we employed a pulse labeling strategy with methionine analog HPG and Click-iT chemistry to follow the translation activity inside the mitochondria of cells treated with CHX, which blocks cytosolic translation.^28,29^ The cells from YPG culture showed about 8 times higher HPG signal than the counterpart from YPD culture (Figures 2B–2D). Similar results were observed using the orthogonal approach with mtDNA-encoded GFP_1–10_ from BiG Mito-Split-GFP in the absence of CHX (Figures 2E–2H).

To rule out the possibility that the significantly higher HPG signal and Mito-Split-GFP abundance in YPG-cultured cells were simply due to increased mitoribosome abundance, we quantified the abundance of MRPs and mt-rRNAs in cells cultured in YPG and YPD. We imaged and quantified 54 endogenously GFP-tagged MRPs, which are functional as shown by normal cell growth and morphology in YPG for imaging (Figures S2D and S2E). The abundance of these 54 MPRs varied, consistent with previous observations that the stoichiometry of different ribosomal proteins (RPs) is not uniformly 1:1.^38,39^ On average, MRP abundance per cell differed by approximately 2-fold between YPG and YPD cultures (Figure 2I). This difference is likely a result of combined transcriptional and post-transcriptional regulation driven by metabolic signals between the two growth conditions (Figures S2F and S2G). The 2-fold MRP abundance difference aligns with the observed 2.5-fold increase in mt-rRNA abundance in mitochondria purified from YPG-cultured cells compared with YPD-cultured cells (Figures 2J and S2H). This 2–2.5-fold higher abundance of G-mitoribosome compared with D-mitoribosome sharply contrasts with the 8-fold increase in translation activity observed in cells cultured in YPG versus YPD media. These results demonstrate that each G-mitoribosome is 2–3 times more productive than the D-mitoribosome, highlighting a significant enhancement in mitochondrial translation activity under respiratory conditions.

### The G-mitoribosome-specific MRPs control translation activity and metabolism of mitochondria

Next, we asked whether these G-mitoribosome-specific MRPs (mS47, mS46, and bL35m, collectively referred to as MRPs^G^) are necessary and sufficient to modulate mitoribosome activity. Knocking out MRP^G^ resulted in poor growth in glycerol medium but exhibited normal growth in glucose medium, confirming their importance for cells to thrive under respiring conditions (Figure S2I). A conventional explanation is that although the absence of MRPs^G^ disrupts mitoribosome function, these fermenting cells in glucose medium are not sensitive to dysfunctional mitoribosomes, as these cells primarily derive their energy from glycolysis. Surprisingly, an mtDNA-encoded GFP_1–10_ reporter revealed that these knockout cells maintained normal levels of translation activity in glucose medium, suggesting that D-mitoribosome function and translation activity remain unaffected by the absence of these MRPs^G^ (Figures S2J–S2L). This also suggests that the minor population of D-mitoribosomes carrying mS47 is not responsible for the mitochondrial translation activity under glucose conditions. By contrast, knocking out these MRPs^G^ significantly reduced the translation activity in cells cultured in glycerol medium, in agreement with the presence of these MRPs^G^ in the G-mitoribosome structure and the poor growth of MRPs^G^ knockout cells in glycerol medium (Figures S2I and S2M–S2O). Notably, despite reduced activity, these mutant mitoribosomes remain functional and continue to translate proteins in glycerol medium (Figures S2M–S2O). This demonstrates that the absence of MRPs^G^ incorporation results in a functional, albeit less active, mitoribosome, consistent with the previous studies.^40–42^ A previous study reported that deletion of bL35m had a negligible impact on mitochondrial respiration and translation.^41^ This discrepancy may be attributed to differences in genetic background—S288c in our study versus W303 in the previous one, which differ by ~15% of the genome—as well as the ectopic expression of RNR1 in the bL35m deletion strain used in that study. Nevertheless, metabolism-dependent antibiotic resistance was also observed in the W303 background (Figures S2P versus S1N). In addition, under fermentative growth, deletion of bL35m only modestly reduced mtDNA abundance (Figure S2Q), suggesting that the impact of bL35m loss on mtDNA maintenance is minimal under this condition. Therefore, MRPs^G^ are essential for assembling a mitoribosome with enhanced translation activity in cells cultured under respiration conditions but are dispensable for the normal activity of mitoribosomes in fermenting cells, which normally have lower mitochondrial translation activity.

**Figure 3.**
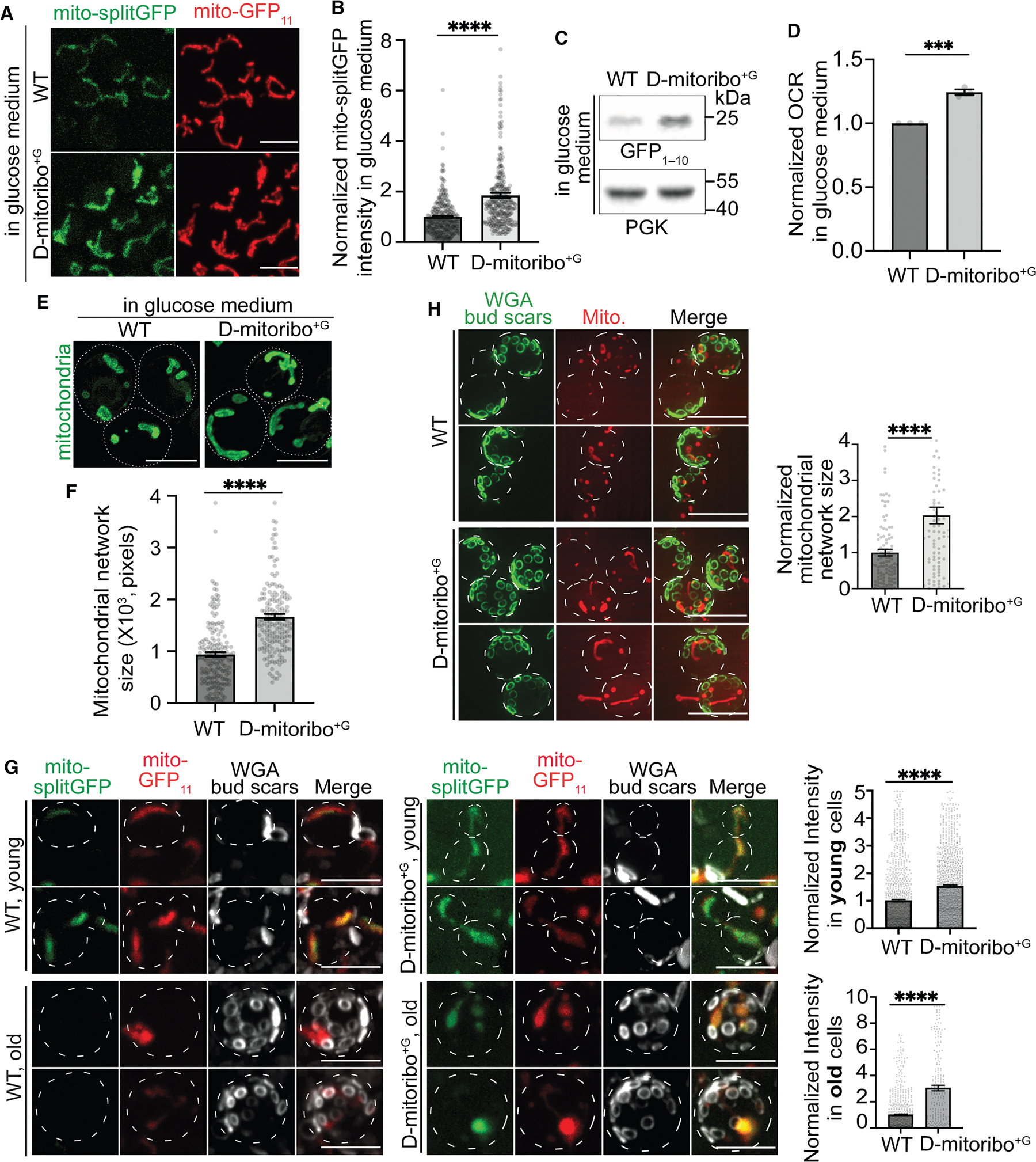
The G-mitoribosome-specific MRPs are sufficient to modulate mitochondrial translation activity and metabolism (A–C) Representative images, quantification, and immunoblot of BiG Mito-Split-GFP in cells cultured in glucose medium, with or without co-expression of three MRPs^G^ from the leakage expression of the Z3EV promoter (D-mitoribosome^+G^ cells). (D) Comparison of the oxygen consumption rate (OCR) between wild-type cells and D-mitoribosome^+G^ cells in glucose medium. (E and F) Representative images and quantification of mitochondrial biomass in wild-type cells and D-mitoribosome^+G^ cells cultured in glucose medium. (G) Representative images and quantification of BiG Mito-Split-GFP in different ages of wild-type cells and D-mitoribosome^+G^ cells cultured in YPD. Yeast bud scars (replicative age) were stained by 633 wheat germ agglutinin (WGA). Individual channels of WGA, BiG Mito-Split-GFP, and mito-GFP_11_-mCherry are merged and displayed as a composite. (H) Representative images and quantification of the mitochondrial network size in old wild-type cells and old D-mitoribosome^+G^ cells cultured in glucose medium. Mitochondria were stained by MitoTracker, and bud scars (replicative age) were stained by AlexaFluor 488-WGA. Individual channels of WGA and MitoTracker are merged and displayed as composite. Bar graphs are mean and SEM (cell numbers for each strain refer to Table S2). Scale bar: 5 μm. WT, wild-type cells.

These MRPs^G^ are expressed at higher levels in glycerol condition compared with glucose culture (Figure S3A). To test whether these MRPs^G^ are sufficient to modulate mitoribosome translation activity, an extra copy of these three MRPs^G^ under the Z3EV promoter was introduced into wild-type cells to co-express and increase the abundance of these MRPs in cells cultured in glucose medium through the leakage expression of the Z3EV promoter (Figure S3B).^43^ Co-immunoprecipitation (coIP) confirmed that these ectopically OE MRPs^G^ were incorporated into D-mitoribosomes without increasing the abundance of other MRPs (Figures S3C–S3E). These modified D-mitoribosomes are referred to as D-mitoribosome^+G^ in the following text. Interestingly, D-mitoribosome^+G^ exhibited increased sensitivity to CAP than wild-type D-mitoribosome, suggesting that the incorporation of these MRPs^G^ also affects the structure of the rRNA (Figure S1R). HPG pulse labeling and the mtDNA-encoded GFP_1–10_ reporter revealed that D-mitoribosome^+G^ was approximately two times more productive than D-mitoribosome in wild-type cells, even though both were cultured in glucose (Figures 3A–3C, S3F, and S3G). This was not due to extra-ribosomal functions of MRPs^G^, as overexpressing individual MRPs^G^ had limited or no effect on mitochondrial translation activity (Figures S3H–S3K). Together, these results demonstrate that the G-mitoribosome-specific MRPs are both necessary and sufficient to modulate mitoribosome activity.

We next investigated whether the enhanced translation activity of D-mitoribosome^+G^ is physiologically relevant and impacts mitochondrial functions. These glucose-cultured cells with D-mitoribosome^+G^ exhibited a significantly increased mitochondrial respiration compared with control cells with D-mitoribosome (Figure 3D). This increased mitochondrial respiration was unexpected, given that the mitoribosome translates only seven subunits of the OXPHOS complexes, while most components of these complexes, along with other mitochondrial enzymes involved in respiration, are encoded by nuclear DNA and translated in the cytosol. The enhanced mitochondrial respiration in D-mitoribosome^+G^ cells suggests that the enhanced translation activity of the mitoribosome orchestrates an increase in the biogenesis of many mitochondrial proteins encoded by nuclear DNA. Indeed, D-mitoribosome^+G^ cells cultured in glucose exhibited significantly increased biomass of mitochondrial network (Figures 3E and 3F). This increased mitochondrial biomass was associated with higher abundance of mitochondrial proteins across all four sub-organellar compartments, including numerous nuclear DNA-encoded OXPHOS components and TOM-TIM complex subunits (Figures S3L and S3M). The elevated levels of TOM-TIM complexes suggest that these cells are primed to import additional mitochondrial proteins synthesized in the cytosol. Knocking out Tpk1/3 of the cytosolic PKA signaling pathway reduces the mitochondrial biogenesis effect of D-mitoribosome^+G^ (Figures S3N and S3O).^44^ This suggests that D-mitoribosome^+G^ sends retrograde signals via Tpk1/3 to the nucleus to regulate mitochondrial biogenesis programs. Collectively, these findings demonstrate that enhancing mitochondrial translation can increase mitochondrial respiration and biogenesis in glucose-cultured cells. Conversely, knocking out these G-mitoribosome-specific MRPs reduces both mitochondrial translation and mitochondrial biogenesis in glycerol-cultured cells (Figures S2M–S2O, S3P, and S3Q).

Given this unexpected role of the MRPs^G^ in regulating mitochondrial translation, respiration, and biogenesis, we investigated whether these MRPs^G^ can suppress age-associated mitochondrial dysfunction—a conserved hallmark of aging resulting from a decline in mitochondrial biogenesis.^45^ We observed reduced mitochondrial translation activity during the replicative aging of wild-type cells cultured in glucose medium (Figure 3G). By contrast, the age-matched D-mitoribosome^+G^ cells maintained significantly higher mitochondrial translation activity during aging under the same conditions (Figure 3G). Consistent with the observations in young cells, aged D-mitoribosome^+G^ cells also exhibited increased mitochondrial biomass compared with the old wild-type cells (Figure 3H). These results suggest that MRPs^G^ are critical and sufficient to prevent mitochondrial biogenesis defects during aging.

### Origins of structural differences between D- and G-mitoribosomes

**Figure 4.**
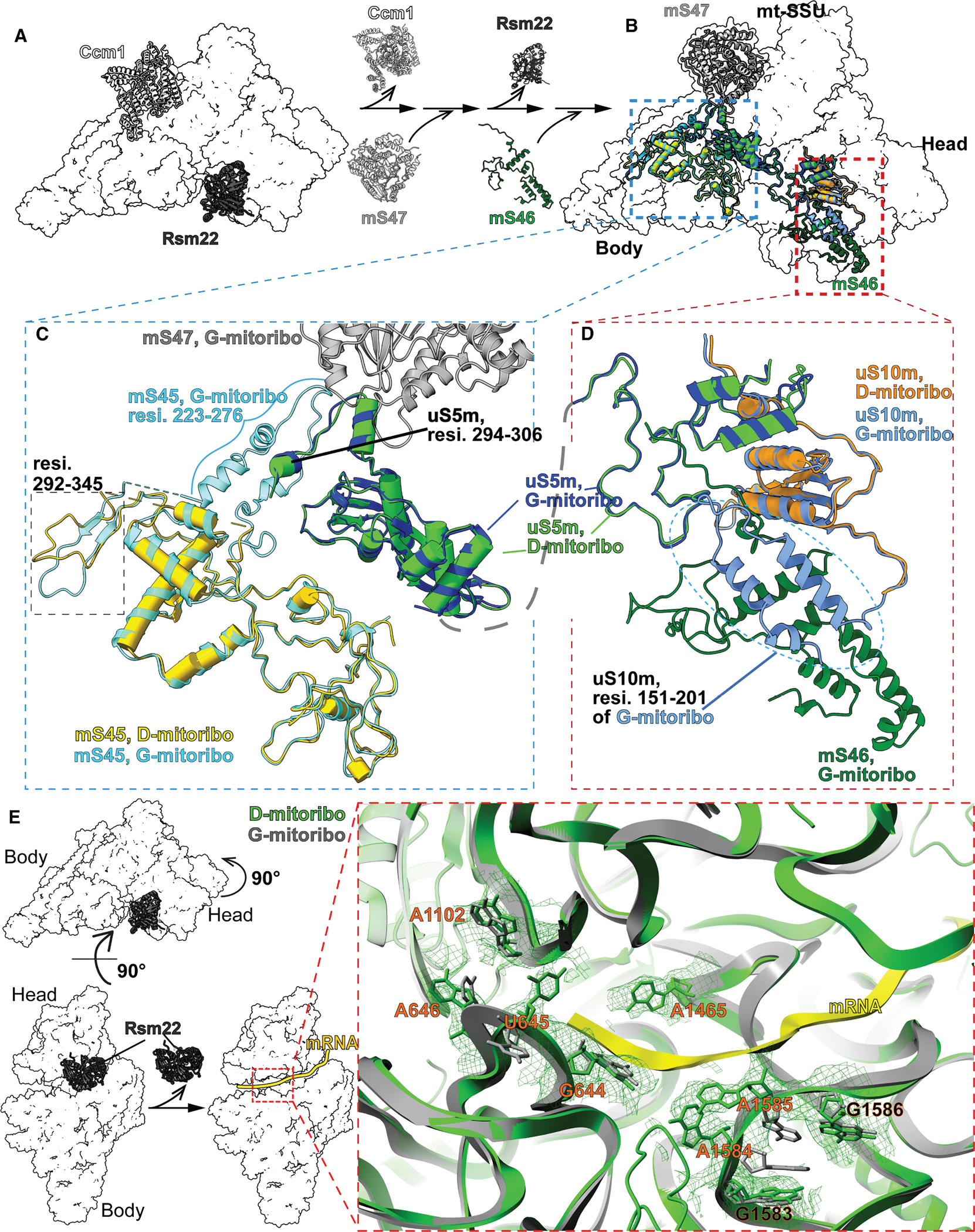
Metabolism-modulated mitoribosome assembly induces conformational differences in MRPs and rRNA (A) Sequential assembly pathway during mitoribosome maturation: chaperone Ccm1 is released from the assembly intermediate, facilitating the insertion of mS47. This is followed by the dissociation of Rsm22 and the subsequent incorporation of mS46.^46^ Although G-mitoribosome assembled in respiring cells incorporates mS47 and mS46, fermenting cells assemble D-mitoribosome without inserting these two subunits. (B–D) Overview of the localization (B) and conformation of mS45 (C), uS5m (C), and uS10m (D) in mature D- and G-mitoribosomes. See also Video S4. (E) Comparison of the conformations of nucleotides near the decoding center reveals key differences between D- and G-mitoribosomes. Rsm22 release is known to induce the folding changes of these nucleotides.^46^ See also Figure S5B for comparison with the assembly intermediate.

Next, we sought to elucidate the origins of the structural and compositional disparities between D- and G-mitoribosomes. We hypothesized that these widespread differences across the MRPs and rRNA result from context-dependent assembly of mitoribosomes. Notably, a detailed Ccm1-mS47-Rsm22-uS10m-mS46 assembly pathway has recently been reported for the late steps of mitoribosome mtSSU biogenesis in respiring glycerol medium.^46^ This pathway suggests that during the late stage of mtSSU biogenesis, the release of chaperone Ccm1 permits the removal of the 5′ end precursor and nucleotides 8–12 of the 15S rRNA and the insertion of mS47, which stabilizes the 223–276 residues of mS45. The replacement of Ccm1 by mS47 then triggers the dissociation of another chaperone, Rsm22, followed by conformational changes in the C-terminal peptide of uS10m, allowing the insertion of mS46.^46^ This ultimately changes the conformation of the uS10m C terminus to lock mS46 inside the mtSSU of the G-mitoribosome (Figures 4A and S4A, glycerol pathway; Video S2).

The Ccm1-mS47-Rsm22-uS10m-mS46 assembly pathway highlights that the compositional and structural differences between the mature D- and G-mitoribosomes are not random but are instead intricately coordinated, reflecting an underlying internal connection originated from these biogenesis steps (Figures 4A and 4B; Video S2). The D- and G-mitoribosomes share the biogenesis steps, including the removal of the 5′ end precursor, the replacement of nucleotides 8–12 of 15S rRNA by the C-terminal residues 294–306 of uS5m, and the maturation of multiple rRNA helices (Figures S4B and 4C; Video S3). However, D-mitoribosome differs from G-mitoribosome in the late assembly steps in that D-mitoribosome skips the incorporation of mS47 and mS46, leading to multiple conformational differences across the entire mtSSU. The lack of mS47 incorporation in the D-mitoribosome prevents the stabilization of the 223–276 residues of mS45 in D-mitoribosome (Figures 4C, S4C, and S4D; Videos S2 and S4). The lack of mS47 incorporation during D-mitoribosome mtSSU assembly likely also affects the downstream insertion of mS46 in D-mitoribosome, based on the described sequential assembly mechanism.^46^ This is probably because uS5m spans across the body-head domain of the mtSSU, with its N terminus wrapping around uS10m and interacting with mS46, while its C terminus interacts with mS47/mS45 (Figures 4C and 4D). Since the mS46 insertion changes the conformation and stabilizes uS10m’s C terminus,^46^ the lack of mS46 insertion in D-mitoribosome prevents the resolution of uS10m’s C terminus by cryo-EM (Figures 4D, S4A, S4E, and S4F; Videos S2 and S4). In addition, skipping the insertion of mS47 and mS46 during the assembly of D-mitoribosome also leads to conformational changes in residues 292–345 of mS45, chain cc (an unknown MRP in G-mitoribosome), residues 311–319 of mS35, and residues 30–78 of Var1 in D-mitoribosome that are different from both assembly intermediates and G-mitoribosome (Figures S4G and S4H).

These differential MRP installations during the assembly of D- and G-mitoribosomes likely cause some rRNA structural differences between D- and G-mitoribosomes, as many rRNA regions fold and mature following the insertion of these MRPs.^46^ This is consistent with the observation that D-mitoribosome^+G^ exhibited increased sensitivity to CAP than wild-type D-mitoribosome, suggesting that the incorporation of these MRPs^G^ converts the PTC regions—where CAP is known to interact—into a conformation resembling that of G-mitoribosome (Figure S1R). Moreover, while most of the 15S rRNA helices mature similarly from assembly intermediates to D- and G-mitoribosomes (Figure S4B), we identified several conformational differences in the 15S rRNA between D- and G-mitoribosomes (Figures 4E and S5A–S5D). For instance, three regions (A1102/G644–A646, A1584–1585, and A1645) of the decoding center, which form a triangular structure that encircles the mRNA, adopt distinct conformations in D-mitoribosome compared with G-mitoribosome (Figures 4E and S5A). Consistent with the role of these MRP installations in rRNA folding, these decoding center regions adopt distinct immature conformations in the assembly intermediates lacking these MRPs, differing from those observed in both D- and G-mitoribosomes (Figure S5B). This suggests that those decoding center regions undergo folding and maturation following the release of Rsm22 and the incorporation of mS46.^46^ Similarly, the absence of bL35m during the maturation of the D-mitoribosome mtLSU may contribute to the distinctive structures observed in the P loop (H80) and the PTC loops, as the incorporation of its human homolog, bL35, precedes the folding of the P loop (H80) and the PTC loops in mitoribosome biogenesis.^47^

### Mechanistic model

**Figure 5.**
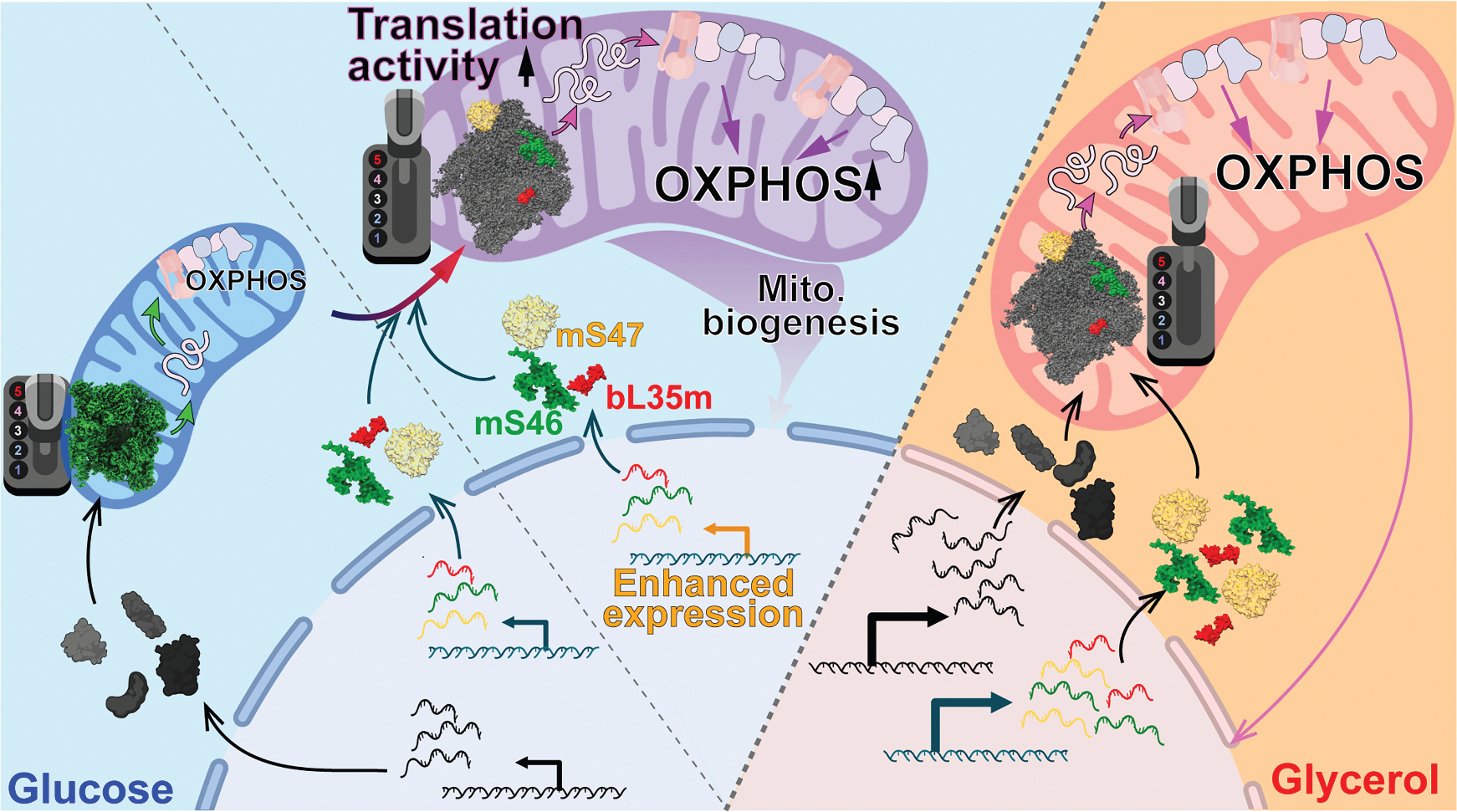
Model of mitochondrial ribosome remodeling in response to metabolic state to regulate translation and biogenesis Cells grown in glucose or glycerol medium express different levels of MRPs and assemble mitoribosomes with different compositions and structures due to the incorporation of three additional MRPs (mS46, mS47, and bL35m). These proteins enhance translation activity, increasing the production of OXPHOS complexes and improving respiration. Upregulating the expression of these MRPs enables their incorporation into D-mitoribosomes, enhancing mitoribosome activity in glucose conditions. This enhanced activity feeds back to the nucleus to increase mitochondrial biogenesis.

Our findings reveal unexpected modifications in the structure and activity of mitoribosomes in response to metabolic environment (Figure 5). Under non-fermentable carbon sources, mitochondria incorporate mS47, mS46, and bL35m into the G-mitoribosome, assembling a highly active ribosome complex that enables cells to thrive under respiring conditions. By contrast, during glucose fermentation, the maturation of the D-mitoribosome follows a distinct pathway in the late assembly steps, bypassing the incorporation of mS47 and mS46 in the mtSSU and bL35m in the mtLSU. This results in a structurally distinct but functional D-mitoribosome with reduced activity, likely due to the different folding and maturation of key rRNA regions, including the decoding center and PTC, which happen after the installation of these MRPs^G^ during mitoribosome assembly. Removing these G-mitoribosome-specific MRPs from the G-mitoribosome reduces its translation activity, whereas increased expression and incorporation of these MRPs in the D-mitoribosome enhances its translation activity. This MRPs^G^-dependent regulation of mitoribosome activity plays a critical role in mitochondrial biogenesis: the absence of MRPs^G^ blocks mitochondrial biogenesis induced by non-fermentable carbon sources. Conversely, their increased expression promotes mitochondrial biogenesis even under glucose fermentation conditions, which typically suppress mitochondrial biogenesis. The observation that enhanced mitoribosome activity induces not only OXPHOS proteins but also other mitochondrial proteins suggests a mitoribosome activity-dependent regulatory mechanism of nuclear mitochondrial biogenesis programs.

## DISCUSSION

Compared with the robust and extensive cytosolic signaling pathways that regulate cytosolic translation activity, mitochondria lack an autonomous signaling network for modulating their own translation activity. The prevailing model posits that mitochondrial translation activity is controlled indirectly by the availability of cytosol-translated OXPHOS subunits, which release chaperone-like mitochondrial translation activators to bind nascent peptides on mitoribosomes.^5,6^ Our findings reveal an alternative regulatory mechanism of mitochondrial translation activity: growth conditions and metabolic contexts can alter the composition and structure of rRNAs and MRPs to adjust the activity of mitoribosomes. This mechanism provides an additional layer of control over mitochondrial protein synthesis, compensating for the absence of a sophisticated translation-modulating signaling network within mitochondria. Importantly, changing mitochondrial translation activity can feed back to control the biogenesis of nuclear-encoded mitochondrial proteins. This mitoribosome activity-dependent regulatory mechanism of nuclear mitochondrial biogenesis programs challenges the conventional view that mitoribosome activity is merely a passive downstream response to the availability of cytosol-translated OXPHOS proteins. Instead, it reveals a dynamic interplay between mitochondrial translation and nuclear gene expression, with mitoribosome activity actively signaling back to the nucleus to modulate the mitochondrial biogenesis programs. Overall, our findings establish that the metabolic environment modulates mitoribosome assembly and structure, providing a noncanonical mechanism for regulating mitochondrial translation activity to align with the metabolic demands for OXPHOS and mitochondrial biogenesis.

Interestingly, although the majority of mitoribosome particles do not incorporate mS47, mS46, and bL35m, these three mitoRPs are still expressed but remain unassembled into the ribosome complex. This suggests that these MRPs may serve mitochondrial functions independent of their role as mitoribosomal components. For example, mS47 is a catalytically active enzyme structurally similar to human 3-hydroxyisobutyryl-coenzyme A (CoA) hydrolase, a mitochondrial protein associated with hereditary mitochondrial disease but not part of the human mitoribosome.^15^ Metabolic signaling pathways are known to induce mitochondrial biogenesis, including MRP expression, upon shifting cells from glucose to glycerol. The skewed correlation between MRP transcripts and protein abundance indicates that additional post-translational regulatory mechanisms control MRP supply, which influences mitoribosome assembly and activity. Future studies are needed to elucidate how environmental cues regulate MRP availability and the mitoribosome assembly pathway to modulate translation activity, as well as to define the mechanisms underlying the retrograde signaling from the mitoribosome to the nuclear mitochondrial biogenesis program.

### Limitations of the study

In this study, we proposed a detailed origin of the compositional and conformational changes in the mtSSU related to mS47 and mS46, informed by a recently published mtSSU assembly pathway. Although we drew on insights from the human mtLSU, it remains unclear how the absence of bL35m incorporation during mtLSU assembly affects the folding of the PTC and P loop, as the assembly pathway in yeast remains uncharacterized and reference structures of assembly intermediates are currently lacking. These limitations preclude a direct structural comparison similar to what we performed for the mtSSU. Consequently, the origin of the compositional and conformational changes in the mtLSU of D- and G-mitoribosomes remains unresolved. Future structural characterization of mtLSU assembly intermediates will be essential to address this question. In addition, the mitoribosome structures and functions analyzed in this study are based on S288c-derived strains. Future work will be needed to determine whether the substantial genetic differences between S288c and W303 contribute to variations in mitoribosome structure and functions.

## RESOURCE AVAILABILITY

### Lead contact

The lead contact is Dr. Chuankai Zhou (kzhou@buckinstitute.org).

### Materials availability

Further information and requests for resources and reagents should be directed to and will be fulfilled by the lead contact.

### Data and code availability

- The cryo-EM maps have been deposited in the Electron Microscopy Data Bank under EMDB: EMD-42720 and EMD-42687. The atomic models have been deposited in the Protein Data Bank under PDB: 8UXA and 8UX4. RNA sequencing data have been deposited in NCBI’s Gene Expression Omnibus (GEO) under accession number GEO: GSE301747, with raw FASTQ data available through the Sequence Read Archive (SRA) under accession number SRA: PRJNA1285088. In addition, the maps/models as well as other raw data have been deposited in Mendeley Data and can be accessed with this link Mendeley Data: https://data.mendeley.com/preview/by7j6d4vmx?a=9030f211-e554-4eb5-b115-9bac393f42b9.
- This paper does not report original code.
- Any additional information required to reanalyze the data reported in this paper is available from the lead contact upon request.

## STAR★METHODS

### EXPERIMENTAL MODEL AND STUDY PARTICIPANT DETAILS

#### Yeast strains and reagents

All the yeast strains used in this study are based on the BY4741 strain background. The GFP-tagged strains used for imaging were from the GFP collection.^49^ All new strains were constructed based on PCR mediated homologous recombination^53^ and genotyped with colony PCR to confirm correct genetic modification. The flag-tagged strains were either generated by PCR amplification of the 3xFlag cassette from tagging plasmids based on pFA6a^54^ or swapped from the GFP library strain directly using the swap plasmid and primers. Ectopic expression of protein was achieved by integrating the linearized plasmids into the TRP1 locus. All plasmids were constructed based on Gibson assembly with Gibson Assembly Master Mix (New England Biolabs, E2611S). The GFP nanobeads were prepared in our lab. The strains, plasmids, primers, antibodies, and chemical reagents used in this study are listed in key resources table.

Yeast cells were grown in YEP with 2% dextrose (YPD) or 2% glycerol (YPG) for over 18 hrs at the indicated temperature. Fresh YPD was used to refresh the glucose culture before imaging. The OD_600_ of cell culture was maintained between 0.4–1.0 overnight and throughout the experiments to avoid metabolic shifts caused by glucose exhaustion. For GFP strains cultured for imaging, 6 ml of cell culture in a 25 ml tube was grown in a rotator drum with constant speed at the indicated temperature. For the cell culture for mitoribosome complex purification, yeast cells were cultured in flask and grown in shaker with a shaking speed at 220 rpm. Yeast cells were harvested, and the pellets were flash frozen in liquid nitrogen and kept at −80 °C before the purification. Similarly, the culture was also refreshed and maintained mid-log OD as above before harvesting.

All mediums used in the study were prepared by autoclaving the yeast extract and peptone for 20 min before adding the filtered carbon source as indicated.

All images were processed by Image J or Fiji software (NIH, Bethesda, MD). For visualization purposes, images were scaled with the bilinear interpolation for figures. The software and algorithms used in this study are listed in key resources table.

### METHOD DETAILS

#### Confocal microscopy

All images other than the super-resolution images and aged cell images were acquired using a Carl Zeiss LSM-510 Confocor 3 system (equipped with two single-photon sensitive APD detectors) with a 100× 1.45 NA Plan-Apochromat objective and a pinhole of one airy unit. The system was driven by Carl Zeiss AIM software for the LSM 510. 488/561 nm excitation was used to excite GFP/RFPs, and emission was collected through the appropriate filters onto the single photon avalanche photodiodes on the Confocor 3. All GFP images were acquired through a 500–550 nm filter. RFP images were acquired with a 580nm long pass filter on the Confocor 3.

The aged cell images were acquired using a Nikon CSU-W1 SoRa spinning disk confocal microscope system equipped with a 100× 1.49 NA Plan-Apochromat objective and a 60× 1.27 NA Plan-Apochromat objective. The Z direction motion is controlled by a Piezo Z stage.

All the cells other than the MitoTracker^™^ stained samples and aged cells were imaged on the slide with coverslip. The MitoTracker^™^ stained cells and aged cells are imaged in 384 well glass plate (Cellvis, P384–1.5H-N, US).

#### GFP strain imaging under different conditions

GFP strains from the GFP library (−80 °C) were refreshed onto YPD plates. The liquid culture was prepared from the fresh yeast plate with 6 ml medium in a 25 ml glass tube and cultured in a rotator drum. The cells in YPD were cultured for more than 14 hrs before refreshing with fresh medium, and the cells in YPG were cultured for more than 20 hrs due to its slower growth. The OD_600_ of cell culture was maintained below 1.0 to ensure sufficient nutrient availability in the medium and consistent metabolism across the cell cultures, as glucose depletion is known to cause metabolic shift. GFP-tagged MRP genes that are malfunctional and cannot grow well in YPG are removed from the comparison.

#### Mitochondrial isolation

*Saccharomyces cerevisiae* strain (bL17m-3C-GFP) was refreshed on YPD plate 2 days ahead at 30 °C. The OD_600_ of the cell culture grown in YPD was kept under 1.0 throughout the experiment (including overnight) to avoid metabolic shift to respiration. Prior to harvesting, the culture was refreshed once with fresh YPD medium for 2 to 4 hrs. Cells cultured in YPG were harvested until an OD_600_ of 1.5 was reached. Cells were harvested at 3,500 g for 5 min, and washed once with 100 ml ddH_2_O. After washing, cells were incubated with 0.1M Tris-SO_4_ (pH 9.5) with 10 mM DTT for 5 min at room temperature, then the cells were pelleted and washed in digestion buffer (20 mM K_2_HPO_4_-HCl, pH 7.4, 1.2 M sorbitol). The cell pellet was resuspended in Lallzyme MMX (Lallemand Inc.) solution (4 g Lallzyme per gram of cells) and digested at room temperature with slow shaking at 100 rpm for 15 min. The cells were then pelleted at 3,000 g for 5 min and washed twice with pre-chilled digestion buffer. All subsequent procedures were carried out at 4 °C.

The cells were resuspended in the homogenization buffer (20 mM HEPES-KOH, pH 7.5, 0.6M sorbitol, 1 mM EDTA). The cell clusters were disassociated thoroughly by applying the cell suspension through a 10μm PTFE syringe filter (Tisch Scientific, SPEC18230, US). The well-separated cells were then lysed by filtering the cells through the 5 μm PTFE syringe filters (Tisch Scientific, SF15015, US). All the lysates were collected and pelleted at 2,000 g for 10 min to remove the intact cells or other organelles from lysate. The supernatant was collected and further centrifuged at 4,500 g for 10 min to remove large cell debris. The supernatant was then centrifuged at 13,000 g for 10 min to pellet the crude mitochondria. The crude mitochondria were resuspended with SEM buffer (250 mM Sucrose, 20 mM HEPES-KOH, pH 7.45, 1 mM EDTA). The crude mitochondria were further purified by centrifuging with a 15%–60% sucrose gradient at 41,000 rpm for 1 hr at 4 °C. The mitochondria located between 32% to 60% sucrose were collected and snap frozen in liquid N_2_. The purified mitochondria were stored at −80 °C.

#### Purification of mitoribosome

Cytosolic ribosomes cause significant contamination in mitochondrial ribosome purification due to low mitochondrial ribosome abundance in YPD. To minimize the cytosolic ribosome contamination, mitoribosomes from YPD culture were purified based on the affinity purification. In brief, the purified mitochondria from above protocol (stored at −80 °C) were equalized on ice and washed twice by SEM buffer (250 mM Sucrose, 20 mM HEPES-KOH, pH 7.45, 1 mM EDTA). The mitochondria pellet was further solubilized with 3 volume of cold mitochondria lysis buffer (25 mM HEPES-KOH pH 7.5, 100 mM KCl, 25 mM MgOAc, 1.7% Triton X-100, 2 mM DTT with 100 ug/ml Chloramphenicol, 100 ug/ml Erythromycin) with gentle mixing immediately. The lysate was gently mixed for 20 min at 4 °C to improve the mitochondria lysis. The lysate was further cleared by centrifuging at 30,000 g for 20 min to remove the membrane fraction. The supernatant was collected and incubated with the GFP nanobody beads for 2 hrs at 4 °C. The collected beads were washed 3 times with washing buffer (25 mM HEPES-KOH, pH 7.5, 100 mM KCl, 25 mM MgOAc, 1.7% Triton X-100, 100 ug/ml Chloramphenicol, 100 ug/ml Erythromycin) and once with washing buffer without Triton. To remove the tag, 0.8–1.2 mg/ml HPV-3C protease (Millipore Sigma, SAE0045, US) was applied to the GFP nanobody beads and the digestion was performed at 4 ° C for 3 hrs. After brief centrifugation, the digest was applied to the Amicon Ultra-0.5 Centrifugal Filter Unit (30 kDa) (EMD Millipore, UFC503096, US) and centrifuged at 14,000 g to a final volume of ~20 ul. The mitoribosome concentration was estimated by measuring A260 with Nanodrop (Thermo Scientific, US).

#### Amine functionalized GO grids preparation

Preparations and functionalization of Graphene Oxide (GO) grids were performed according to published protocol.^55,56^ Briefly, an epoxy coated stainless steel mesh stand was placed at the bottom of a glass petri dish filled with DI water. 300 Mesh, R1.2/1.3 Au Quantifoil grids were placed on the mesh stand with carbon side facing upward. The GO solution was spread onto the water surface with a syringe. Then the water in the dish was removed and the GO coated grids were allowed to dry at the room temperature. These dried GO-covered grids were then submerged in 10 mM ethylenediamine solution diluted in dimethyl sulfoxide and incubated for 5 hrs at room temperature, followed by washing twice with DMSO, twice with autoclaved water, twice with ethanol, and dried under ambient conditions. These modified grids were stored dry at −20 °C until use.

#### Electron microscopy sample preparation and data collection

Freshly prepared mitoribosome was first checked with negative staining. Negative staining of the mitoribosome was done with 0.75% uranium formate according to published protocol.^57^ Grids were examined using an FEI T12 microscope at 120 kV, and images were collected using a 4k × 4k charge-coupled device (CCD) camera (UltraScan 4000, Gatan).

##### Prepare cryo-grids

Fresh mitoribosome samples (2.5μl) were applied onto the amine functionalized Graphene Oxide grids, and then blotted with No.1 blotting filter paper from TED PELLA for 4 s (blotting force was preset to 0), followed by plunge-frozen in liquid ethane cooled by liquid nitrogen using a Thermo Fisher Vitrobot IV (sample chamber set at 22°C and 100% humidity).

##### Data collection

Grids were examined and screened using an FEI Arctica Talos at 200 kV with a Gatan K3 camera. The grids with high quality ice and mitoribosome particles were used for data collection on a Titan Krios at the UCSF Cryo-EM Center for Structural Biology operated at an acceleration voltage of 300 kV and equipped with a BioQuantum energy filter (slit width set to 20 eV) and a K3 direct electron detector (Gatan).

Cryo-EM datasets were collected using SerialEM.^58^ Multishot collection (3×3 arrays) was performed with beam-tilt compensation and a maximum image shift of 3.5μm. All images were acquired with a nominal magnification of 105k, resulting in a physical pixel size of 0.835Å. Defocus range was set from −0.8 μm to −2.0 μm. A total of 16,372 images were collected, each was dose-fractionated to 117 movie frames with a total exposure time of 5.9 s, resulting in a total fluence of ~67 electrons per Å^2^.

#### Cryo-EM data analysis

All the movies were motion corrected by MotionCorr2,^59^ followed by manual inspection to exclude bad micrographs that are not suitable for further processing. CTF determination was performed by Ctffind4^60^ which is incorporated in RELION4.0.^61^ 9,126 micrographs with the good quality were kept for further analysis, from which 485,179 picks were autopicked by RELION 4.0 laplacian autopicking at default settings and with the picking threshold at 0.05. Extracted particles were subjected to multiple runs of 2D classifications with a 360Å-diameter mask and regularization parameter T at 2 to exclude the junk particles and LSU only particles. 277,072 particles with a rough shape of ribosome were pooled together for 3D classification into 4 classes with a general mask at 180 Å and T regularization value at 4. The initial reference map was obtained by low pass filtering the PDB 5MRC to 30 Å. Only one resulting class of 54,042 particles from 3D classification shows a shape of intact ribosome, which were further refined in Relion4.0, followed by 3D classification. This resulted in two groups of particles, later named as D-mitoribosome class I (12,907 particles) and D-mitoribosome class II (24,966 particles). Particles of D-mitoribosome class II were further subjected to one more round of 3D classification without alignment, yielding 14,543 particles for 3D high-resolution reconstruction of class II D-mitoribosome. Final reconstructions of D-mitoribosomes were calculated by using cisTEM.^62^ Resolutions were estimated using the gold standard Fourier shell.^63^

##### Focused 3D classification

To explore the mitochondrial ribosome heterogeneity, particles of D-mitoribosome class I and II were further analyzed by focused 3D classifications with a mask of mS47, resulting in three distinct groups that were further refined and reconstructed with cisTEM.

Meanwhile, Global 3D classification was done with CryoDRGN using default parameters.

#### Model building

For the model building, the initial model was generated by fitting the available coordinates into our cryo-EM density maps. These coordinates include the G-mitoribosome (PDB ID 5MRC), rRNA of L7/12 stalk and L1 stalk from PDB ID 6ZSD and 7TOP, and the uL10m and uL11m from Alphafold.^64^ The inconsistent parts were then manually built and refined in coot.^65^ The structures were refined using Phenix^66^ with secondary structure constraints.

#### Mito-FUNCAT assay (Fluorescent noncanonical amino acid tagging for mitochondrial translation)

Yeast cells were cultured overnight in the indicated conditions and refreshed for 2–4 hrs before harvesting. 1 OD_600_ cells were used for the Click-iT mitochondrial translation assay (Mito-FUNCAT). To test the impact of the antibiotics on mitochondrial translation, the cells were incubated with the culture medium including 2 mg/ml Chloramphenicol (CAP), 2 mg/ml Erythromycin (ERY) for 30 min under indicated conditions, respectively. Cells were harvested and washed with SC without methionine and with glucose or glycerol twice and continued to be cultured in the SC without methionine and with glucose or glycerol with 500uM L-Homopropargylglycine (HPG), 100 ug/ml Cycloheximide and antibiotics with the indicated concentrations for another 15min. The cells were collected by centrifuging at 3,000 g for 1 min and washed twice with icy Buffer A (300 mM Sucrose with 10 mM HEPES, 10 mM NaCl, 5 mM MgCl_2_, pH 7.5). Cells were resuspended with icy Buffer A with 1 mg/ml of freshly prepared digitonin and rotated at room temperature for 15 min. After two rounds washing with icy Buffer A, cells were fixed and permeated in the Buffer A with 3.7% Formaldehyde for 15min, following by two rounds washing with icy Buffer A. Click chemistry was performed using the Click-iT^™^ HPG Alexa Fluor^™^ 594 Protein Synthesis Assay Kit (Invitrogen^™^, C10429, US) with azide Alexa Fluor 594. The click reaction was performed in dark for 30 min in the rotator and terminated with two rounds washing of reaction rinse buffer. All the imaging was performed using Zeiss LSM510 and APD (Carl Zeiss, Jena, Germany).

#### Relative rotation analysis of the mtSSU and mtLSU

The 2D rotation plots for backbone phosphorus atoms, alpha carbons, and both together were generated by choosing a plane maximally separating atoms in the SSU and LSU and projecting the body backbone atoms of the SSU onto this plane. For the comparison between D-mitoribosome class I and class II, vectors start at the D-mitoribosome class I backbone positions and extend to the D-mitoribosome class II backbone positions. Colors correspond to 3D distances. A center of rotation was determined by maximizing the tangentiality of the vectors relative to a potential center. In this way, the center of rotation was chosen such that vectors from the center to the D-mitoribosome class I backbone position were maximally orthogonal to vectors from the D-mitoribosome class I to class II backbone positions. Then, an angle of rotation was computed for each backbone atom based on its distance moved in the plane and its distance from the center. For these steps, only backbone displacements over 3 Angstroms were included to avoid spurious shifts.

The implementation of this procedure to analyze the relative rotation of 15s rRNAs or subunits from two structures is available at https://github.com/willnickols/yeast_mitoribosome_viewing.

#### Oxygen consumption rate (OCR) measurement with Seahorse

Cells were cultured overnight in YPD medium to OD_600_ ~0.5 and refreshed the following day for 4–5 hrs. Cell density was determined using Countess^™^ Cell Counting Chamber Slides and the EVE^™^ automated cell counter (NanoEntek, KOR). Around 2 × 10^6^ cells were added into each well that has been pretreated with 80 μL of 0.1 mg/ml poly-L-lysine solution. The plate was centrifuged at 50 g for 3 min before being placed at 30 °C incubator without CO_2_ for 45 min. The assay was performed with a Seahorse XFe24 analyzer and kits (Agilent Technologies^™^, US). The parameters used are: 1) Calibration Equilibration: Yes; 2) Baseline settings: Mix for 2 min, Wait for 0 min, Measure for 2 min; Cycles: 3. Each strain was prepared in triplicate, and the assay was repeated three times in different days.

#### Fluorescent staining

Cells were cultured overnight to OD_600_ ~0.5. About 0.2 OD_600_ cells were collected and washed once in 10 mM HEPES, pH 7.6 with 5% glucose or glycerol. Staining buffer was prepared by mixing MitoTracker^™^ Green FM (Invitrogen^™^, M7514) stock into the same buffer to a working concentration of 100nM MitoTracker^™^ Green FM for cells in glucose medium, and 50nM MitoTracker^™^ Green FM for cells in glycerol medium. For each sample, 1ml of the staining buffer was added and mixed well with the cells. The staining was performed in the dark at room temperature (RT) for 15min. The cells were washed twice with 1ml of 10 mM HEPES, pH 7.6 with 5% glucose or glycerol, and then seeded onto the 384 well glass plate before imaging. Same protocol was used for MitoTracker^™^ Red CMXRos (Invitrogen^™^, M7513) staining, the working concentration for MitoTracker^™^ Red CMXRos in YPD is 100nM.

For Calcofluor white staining, 0.7ul of Calcofluor white (Sigma-Aldrich, 18909–100ML-F) stock (1mg/ml) was added into 1ml of cell culture. The stain was performed at RT for 15min, followed by two washes with SC-Methionine medium for the Mito-FUNCAT assay. For WGA staining, ~0.2 OD_600_ aged cells were washed once with YPD medium and resuspended in 1ml YPD. Then, 5ul of Wheat germ agglutinin (WGA) (Biotium, 29024–1) conjugates stock (0.2mg/ml, in PBS, pH7.2) was added into aged cells suspension. The staining was performed at RT for 30min. The cells were pelleted down for Mitotracker staining.

For DAPI staining, 1ul of DAPI stock (5mg/ml) (Sigma-Aldrich, 10236276001) was added into 1ml of cell culture. The stain was performed at RT for 30min, followed by one wash with PBS buffer.

#### Co-immunoprecipitation (Co-IP)

About 20 OD_600_ cells for ZY6182 and ZY6183 and ~150 OD_600_ for ZY6180 and ZY6181 were collected and washed once with ddH_2_O. The cells were reduced with 1mM DTT in 0.1M Tri-SO_4_ (pH 9.5) for 5min at RT then digested with 1ml of 70 mg/ml Lallzyme MMX (Lallemand Inc.). After washing once with cool digestion buffer, the cells were lysed with 1ml lysis buffer (20 mM HEPES-KOH, pH 7.4, 100mM KCl, 25mM Magnesium acetate, 1% Triton, protease inhibitor cocktail, 1mM PMSF). The cell lysate was kept on ice for 10 min to allow sufficient lysis and then centrifuged at 15,000 g at 4 °C for 10 min. A 50ul aliquot of the supernatant was collected as the input. The remaining supernatant was incubated with 20ul ANTI-FLAG^®^ M2 Affinity Gel (A2220, Sigma). The mixture was rotated for 1 hour at 4 °C. The beads were washed three times with 1ml lysis buffer and once with the wash buffer (20 mM HEPES-KOH, pH 7.4, 100mM KCl, 25mM Magnesium acetate). The beads were resuspended in 50ul of 1x sample buffer and boiled for 5mins to elute the proteins from the beads. The input and elute was subjected to SDS-PAGE and western blot.

#### Aged cells preparation

About 1.5 OD_600_ of yeast cells at mid-log phase were collected and washed three times with PBS buffer (pH 8.0). The cells were then incubated with 0.35mg of NHS-dPEG^®^12-biotin (Sigma, QBD10198–1000MG) at RT for 30 min, followed by three washes with cold PBS buffer (pH 7.2). After washing, the cells were mixed with 15 ul of BioMag streptavidin beads (Polysciences, 84660–5) and incubated for 1 hr at 4 °C. The cells were then cultured in a deep-well plate with medium replacement every 4 hrs for total of 16 hrs. The preparation is performed with Tecan Fluent 480 Liquid Handler.

#### mt-rRNA abundance quantification

About 200 OD_600_ of yeast cells (ZY6184: rpl25–6xHis-KanMX; *trp*::pGDP-preSU9-GFP-APEX2-NatMX) grown in YPD and YPG were collected respectively. Mitochondria from both cell samples were isolated as previously described. The isolated mitochondria were washed 3 times with SEM buffer (250 mM Sucrose, 20 mM HEPES-KOH PH7.45, 1mM EDTA) and completely lysed with the lysis buffer provided in the Quick-RNA Miniprep Kit (Zymo Research, 76020–128). The lysate was then clarified by centrifuging at 15, 000g for 15min. The resulting supernatant was incubated with HisPur^™^ Cobalt Superflow Agarose (Thermo Scientific, PI25228) for 30min to remove cytosolic ribosome contamination, taking advantage of the 6xHis tag on endogenous rpl25. The mt-rRNA and the protein from the mitochondria were purified from the mitochondrial lysate using the Quick-RNA Miniprep Kit. To account for differences in cell size and mitochondrial abundance per cell between the two growth conditions, we normalized the total abundance of pGDP-preSU9-GFP from lysed mitochondria to the abundance per cell under each condition, which allowed for a more accurate estimation of the relative cell numbers. In the end, the mt-rRNA abundance from same number of cells were quantified and compared.

#### RNA-seq sample preparation

Yeast cells were cultured in synthetic complete (SC) medium supplemented with either glucose or glycerol as the sole carbon source. For each sample, 5–10 OD_600_ unit of cells was harvested by centrifugation and washed once with nuclease-free water (ddH_2_O). Cell walls were digested using Lallzyme MX (70 mg/ml) for 3 minutes at room temperature, followed by an immediate wash with ice-cold digestion buffer. Total RNA was extracted using the Quick-RNA Miniprep Kit (Zymo Research, R1054), following the manufacturer’s instructions. RNA concentration and purity were assessed using a NanoDrop 2000c spectrophotometer (Thermo Scientific). RNA library preparation and high-throughput sequencing (PE150, NovaSeq X Plus) were performed by Novogene (USA). Three biological replicates were prepared for each condition.

### QUANTIFICATION AND STATISTICAL ANALYSIS

All experiments were repeated multiple times to confirm reproducibility. Data are representative of at least two independent repeats. All quantifications are presented as the means ± standard error of mean (SEM). The statistical test for each bar graph in the figures was an unpaired two-tailed t-test with Welch’s correction, *p < 0.05; **p <0.01; ***p < 0.001; ****p < 0.0001.

## Supplementary Material

1

2

3

4

5

6

SUPPLEMENTAL INFORMATION

Supplemental information can be found online at https://doi.org/10.1016/j.molcel.2025.10.012.

## Figures and Tables

**KEY RESOURCES TABLE:** 

REAGENT or RESOURCE	SOURCE	IDENTIFIER

Antibodies		

Anti-GFP	Sigma-Aldrich	SAB4301138; RRID:AB_2750576
Anti-GFP, N-terminal antibody	Sigma-Aldrich	G1544; RRID: AB_439690
Anti-FLAG	Sigma-Aldrich	Cat# F1804; RRID:AB_262044
Anti-PGK	Abcam	Cat# ab113687; RRID:AB_10861977

Chemicals, peptides, and recombinant proteins		

3x FLAG peptide	Sigma-Aldrich	F4799-25MG
Anti-FLAG M2 affinity gel	Sigma-Aldrich	A2220
HRV-3C protease	Sigma-Aldrich	SAE0045
Chloramphenicol	Sigma-Aldrich	C0378-25MG
Erythromycin	Sigma-Aldrich	E5389-5G
Cycloheximide	Sigma-Aldrich	C4859-1ML
Click-iT^™^ HPG Alexa Fluor^™^ 594 Protein Synthesis Assay Kit	Invitrogen^™^	C10429
L-Homopropargylglycine (HPG)	Click Chemistry Tools	1067-25
Digitonin	Sigma-Aldrich	D141-100MG
DAPI	Sigma-Aldrich	10236276001
Calcofluor White Stain	Sigma-Aldrich	18909-100ML-F
CF^®^633 Wheat germ agglutinin (WGA) conjugates	Biotium	29024-1
CF^®^488A Wheat Germ Agglutinin (WGA)	Biotium	29022-1
MitoTracker^™^ Green FM	Invitrogen^™^	M7514
MitoTracker^™^ Red CM-H2Xros	Invitrogen^™^	M7513
BioMag^®^ Streptavidin	Polysciences	84660-5
NHS-dPEG^®^12-biotin	Sigma-Aldrich	QBD10198-1000MG
HisPur^™^ Cobalt Superflow Agarose	Thermo Scientific	PI25228
Poly-L-lysine hydrobromide	Sigma-Aldrich	P2636-25MG
LALLZYME MMX	lodi wine labs	60011-07-40

Deposited data		

Raw data	This study	Mendeley Data: https://data.mendeley.com/preview/by7j6d4vmx?a=9030f211-e554-4eb5-b115-9bac393f42b9
RNAseq raw data	This study	**BioProject**: PRJNA1285088;GEO: GSE301747
Cryo-EM maps	This study	Electron Microscopy Data Bank: EMD-42720 and EMD-42687
Atomic models	This study	Protein Data Bank: 8UXA and 8UX4
Validation reports	This study	wwPDB validation reports: DEPOSITION ID: D_1000278977, PASSWORD: rOzVjDElXr

Experimental models: Organisms/strains		

Wild type	This study	N/A
bL17m-3C-GFP-KanMX	This study	N/A
BiG Mito-Split-GFP; *trp::*GAP-preSU9-mCherry-3xGFP β11-NatMX	This study	N/A
sfGFP^m^	Martin Ott lab (MOY1355)^48^	Matα ade2-1 his3-11,15 trp1-1 leu2-3,112 ura3-1, sfGFP^m^::cox2, COX2
Δ*mS46*:KanMX	Yeast Knockout (YKO) Collection	N/A
Δ*mS47*:KanMX	Yeast Knockout (YKO) Collection	N/A
Δ*bL35m*:KanMX	Yeast Knockout (YKO) Collection	N/A
mS46-3C-GFP	This study	N/A
mS47-GFP	Yeast GFP collection^49^	N/A
bL35m-GFP	Yeast GFP collection^49^	N/A
Δ*mS46*:KanMX; BiG Mito-Split-GFP_1-10_; *trp::*pGAP-preSU9-mCherry-3xGFP β11-NatMX	This study	N/A
Δ*mS47*:KanMX; BiG Mito-Split-GFP_1-10_; *trp::*pGAP-preSU9-mCherry-3xGFP β11-NatMX	This study	N/A
Δ*bL35m*:KanMX; BiG Mito-Split-GFP_1-10_; *trp::*pGAP-preSU9-mCherry-3xGFP β11-NatMX	This study	N/A
*trp::*pTDH-NatMX; mS46::pRS416-Z_3_EV-LeuMX-pZ_3_-mS46; mS47::pRS416-Z_3_EV-KanMX-pZ_3_-ms47; bL35m::pRS416-Z_3_EV-hphMX-pZ_3_-bL35m	This study	N/A
BiG Mito-Split-GFP_1-10_; *trp::*pGAP-preSU9-mCherry-3xGFP β11-NatMX; mS46:: pRS416-Z_3_EV-LeuMX-pZ_3_-mS46; mS47::pRS416-Z_3_EV-KanMX-pZ_3_-ms47; bL35m::pRS416-Z_3_EV- hphMX-pZ_3_-bL35m	This study	N/A
pRS416-Z_3_EV-LeuMX-pZ_3_-mS46-yeGFP	This study	N/A
pRS416-Z_3_EV-His3MX-pZ_3_-mS47-yeGFP	This study	N/A
pRS416-Z_3_EV-hphMX-pZ_3_-bL35m-yeGFP	This study	N/A
uS4m-3xFlag-Ura; *trp*::TDH3-NatMX; amp::pRS416-Z_3_EV-LeuMX-pZ_3_-mS46-yeGFP	This study	N/A
bL17m-3xFlag-Ura; *trp*::TDH3-NatMX; amp::pRS416-Z_3_EV-LeuMX-pZ_3_-mS46-yeGFP	This study	N/A
uS4m-3xFlag-Ura; *trp*::TDH3-NatMX; amp::pRS416-Z_3_EV-His3MX-pZ_3_-mS47-yeGFP	This study	N/A
bL17m-3xFlag-Ura; *trp*::TDH3-NatMX; amp::pRS416-Z_3_EV-His3MX-pZ_3_-mS47-yeGFP	This study	N/A
bL17m-3xFlag-Ura; pGAP-preSU9-mCherry-3xGFP β11-NatMX; pRS416-Z_3_EV-LeuMX-pZ_3_-mS46; pRS416-Z_3_EV-His3MX-pZ_3_-ms47; pRS416-Z_3_EV- hphMX-pZ_3_-bL35m	This study	N/A
BiG Mito-Split-GFP_1-10_; pGAP-preSU9-mCherry-3xGFP β11-NatMX; pRS416-Z_3_EV-LeuMX-pZ_3_-mS46	This study	N/A
BiG Mito-Split-GFP_1-10_; pGAP-preSU9-mCherry-3xGFP β11-NatMX; pRS416-Z_3_EV-KanMX-pZ_3_-mS47	This study	N/A
BiG Mito-Split-GFP_1-10_; pGAP-preSU9-mCherry-3xGFP β11-NatMX; pRS416-Z_3_EV-hphMX-pZ_3_-bL35m	This study	N/A
pRS416-Z_3_EV-LeuMX-pZ_3_-mS46-CEN; pRS416-Z_3_EV-His3MX-pZ_3_-ms47-CEN; pRS416-Z_3_EV- hphMX-pZ_3_-bL35m-CEN	This study	N/A
Cox5b-GFP; pRS416-Z_3_EV-LeuMX-pZ_3_-VN-CEN; pRS416-Z_3_EV-KanMX-pZ_3_-VN-CEN; pRS416-Z_3_EV- hphMX-pZ_3_-VN-CEN	This study	N/A
Cox5b-GFP; pRS416-Z_3_EV-LeuMX-pZ_3_-mS46-CEN; pRS416-Z_3_EV-KanMX-pZ_3_-ms47-CEN; pRS416-Z_3_EV- hphMX-pZ_3_-bL35m-CEN	This study	N/A
Cox7-GFP; pRS416-Z_3_EV-LeuMX-pZ_3_-VN-CEN; pRS416-Z_3_EV-KanMX-pZ_3_-VN-CEN; pRS416-Z_3_EV- hphMX-pZ_3_-VN-CEN	This study	N/A
Cox7-GFP; pRS416-Z_3_EV-LeuMX-pZ_3_-mS46-CEN; pRS416-Z_3_EV-KanMX-pZ_3_-ms47-CEN; pRS416-Z_3_EV-hphMX-pZ_3_-bL35m-CEN	This study	N/A
Cox6-GFP; pRS416-Z_3_EV-LeuMX-pZ_3_-VN-CEN; pRS416-Z_3_EV-KanMX-pZ_3_-VN-CEN; pRS416-Z_3_EV- hphMX-pZ_3_-VN-CEN	This study	N/A
Cox6-GFP; pRS416-Z_3_EV-LeuMX-pZ_3_-mS46-CEN; pRS416-Z_3_EV-KanMX-pZ_3_-ms47-CEN; pRS416-Z_3_EV-hphMX-pZ_3_-bL35m-CEN	This study	N/A
ATP4-GFP; pRS416-Z_3_EV-LeuMX-pZ_3_-VN-CEN; pRS416-Z_3_EV-KanMX-pZ_3_-VN-CEN; pRS416-Z_3_EV- hphMX-pZ_3_-VN-CEN	This study	N/A
ATP4-GFP; pRS416-Z_3_EV-LeuMX-pZ_3_-mS46-CEN; pRS416-Z_3_EV-KanMX-pZ_3_-ms47-CEN; pRS416-Z_3_EV- hphMX-pZ_3_-bL35m-CEN	This study	N/A
QCR9-GFP; pRS416-Z_3_EV-LeuMX-pZ_3_-VN-CEN; pRS416-Z_3_EV-KanMX-pZ_3_-VN-CEN; pRS416-Z_3_EV- hphMX-pZ_3_-VN-CEN	This study	N/A
QCR9-GFP; pRS416-Z_3_EV-LeuMX-pZ_3_-mS46-CEN; pRS416-Z_3_EV-KanMX-pZ_3_-ms47-CEN; pRS416-Z_3_EV- hphMX-pZ_3_-bL35m-CEN	This study	N/A
ATP2-GFP; pRS416-Z_3_EV-LeuMX-pZ_3_-VN-CEN; pRS416-Z_3_EV-KanMX-pZ_3_-VN-CEN; pRS416-Z_3_EV- hphMX-pZ_3_-VN-CEN	This study	N/A
ATP2-GFP; pRS416-Z_3_EV-LeuMX-pZ_3_-mS46-CEN; pRS416-Z_3_EV-KanMX-pZ_3_-ms47-CEN; pRS416-Z_3_EV- hphMX-pZ_3_-bL35m-CEN	This study	N/A
TIM23-GFP; pRS416-Z_3_EV-LeuMX-pZ_3_-VN-CEN; pRS416-Z_3_EV-KanMX-pZ_3_-VN-CEN; pRS416-Z_3_EV- hphMX-pZ_3_-VN-CEN	This study	N/A
TIM23-GFP; pRS416-Z_3_EV-LeuMX-pZ_3_-mS46-CEN; pRS416-Z_3_EV-KanMX-pZ_3_-ms47-CEN; pRS416-Z_3_EV- hphMX-pZ_3_-bL35m-CEN	This study	N/A
CCP1-GFP; pRS416-Z_3_EV-LeuMX-pZ_3_-VN-CEN; pRS416-Z_3_EV-KanMX-pZ_3_-VN-CEN; pRS416-Z_3_EV- hphMX-pZ_3_-VN-CEN	This study	N/A
CCP1-GFP; pRS416-Z_3_EV-LeuMX-pZ_3_-mS46-CEN; pRS416-Z_3_EV-KanMX-pZ_3_-ms47-CEN; pRS416-Z_3_EV- hphMX-pZ_3_-bL35m-CEN	This study	N/A
ATP3-GFP; pRS416-Z_3_EV-LeuMX-pZ_3_-VN-CEN; pRS416-Z_3_EV-KanMX-pZ_3_-VN-CEN; pRS416-Z_3_EV- hphMX-pZ_3_-VN-CEN	This study	N/A
ATP3-GFP; pRS416-Z_3_EV-LeuMX-pZ_3_-mS46-CEN; pRS416-Z_3_EV-KanMX-pZ_3_-ms47-CEN; pRS416-Z_3_EV- hphMX-pZ_3_-bL35m-CEN	This study	N/A
TIM50-GFP; pRS416-Z_3_EV-LeuMX-pZ_3_-VN-CEN; pRS416-Z_3_EV-KanMX-pZ_3_-VN-CEN; pRS416-Z_3_EV- hphMX-pZ_3_-VN-CEN	This study	N/A
TIM50-GFP; pRS416-Z_3_EV-LeuMX-pZ_3_-mS46-CEN; pRS416-Z_3_EV-KanMX-pZ_3_ -ms47-CEN; pRS416-Z_3_EV- hphMX-pZ_3_-bL35m-CEN	This study	N/A
LEU9-GFP; pRS416-Z_3_EV-LeuMX-pZ_3_-VN-CEN; pRS416-Z_3_EV-KanMX-pZ_3_-VN-CEN; pRS416-Z_3_EV- hphMX-pZ_3_-VN-CEN	This study	N/A
LEU9-GFP; pRS416-Z_3_EV-LeuMX-pZ_3_-mS46-CEN; pRS416-Z_3_EV-KanMX-pZ_3_-ms47-CEN; pRS416-Z_3_EV-hphMX-pZ_3_-bL35m-CEN	This study	N/A
TOM71-GFP; pRS416-Z_3_EV-LeuMX-pZ_3_-VN-CEN; pRS416-Z_3_EV-KanMX-pZ_3_-VN-CEN; pRS416-Z_3_EV- hphMX-pZ_3_-VN-CEN	This study	N/A
TOM71-GFP; pRS416-Z_3_EV-LeuMX-pZ_3_-mS46-CEN; pRS416-Z_3_EV-KanMX-pZ_3_-ms47-CEN; pRS416-Z_3_EV-hphMX-pZ_3_-bL35m-CEN	This study	N/A
TIM16-GFP; pRS416-Z_3_EV-LeuMX-pZ_3_-VN-CEN; pRS416-Z_3_EV-KanMX-pZ_3_-VN-CEN; pRS416-Z_3_EV- hphMX-pZ_3_-VN-CEN	This study	N/A
TIM16-GFP; pRS416-Z_3_EV-LeuMX-pZ_3_-mS46-CEN; pRS416-Z_3_EV-KanMX-pZ_3_-ms47-CEN; pRS416-Z_3_EV-hphMX-pZ_3_-bL35m-CEN	This study	N/A
TOM70-GFP; pRS416-Z_3_EV-LeuMX-pZ_3_-VN-CEN; pRS416-Z_3_EV-KanMX-PZ_3_-VN-CEN; pRS416-Z_3_EV- hphMX-pZ_3_-VN-CEN	This study	N/A
TOM70-GFP; pRS416-Z_3_EV-LeuMX-pZ_3_-mS46-CEN; pRS416-Z_3_EV-KanMX-pZ_3_-ms47-CEN; pRS416-Z_3_EV-hphMX-pZ_3_-bL35m-CEN	This study	N/A
ATP5-GFP; pRS416-Z_3_EV-LeuMX-pZ_3_-VN-CEN; pRS416-Z_3_EV-KanMX-pZ_3_-VN-CEN; pRS416-Z_3_EV- hphMX-pZ_3_-VN-CEN	This study	N/A
ATP5-GFP; pRS416-Z_3_EV-LeuMX-pZ_3_-mS46-CEN; pRS416-Z_3_EV-KanMX-pZ_3_-ms47-CEN; pRS416-Z_3_EV-hphMX-pZ_3_-bL35m-CEN	This study	N/A
COR1-GFP; pRS416-Z_3_EV-LeuMX-pZ_3_-VN-CEN; pRS416-Z_3_EV-KanMX-pZ_3_-VN-CEN; pRS416-Z_3_EV- hphMX-pZ_3_-VN-CEN	This study	N/A
COR1-GFP; pRS416-Z_3_EV-LeuMX-pZ_3_-mS46-CEN; pRS416-Z_3_EV-KanMX-pZ_3_-ms47-CEN; pRS416-Z_3_EV-hphMX-pZ_3_-bL35m-CEN	This study	N/A
TIM44-GFP; pRS416-Z_3_EV-LeuMX-pZ_3_-VN-CEN; pRS416-Z_3_EV-KanMX-pZ_3_-VN-CEN; pRS416-Z_3_EV- hphMX-pZ_3_-VN-CEN	This study	N/A
TIM44-GFP; pRS416-Z_3_EV-LeuMX-pZ_3_-mS46-CEN; pRS416-Z_3_EV-KanMX-pZ_3_-ms47-CEN; pRS416-Z_3_EV-hphMX-pZ_3_-bL35m-CEN	This study	N/A
TOM20-GFP; pRS416-Z_3_EV-LeuMX-pZ_3_-VN-CEN; pRS416-Z_3_EV-KanMX-pZ_3_-VN-CEN; pRS416-Z_3_EV- hphMX-pZ_3_-VN-CEN	This study	N/A
TOM20-GFP; pRS416-Z_3_EV-LeuMX-pZ_3_-mS46-CEN; pRS416-Z_3_EV-KanMX-pZ_3_-ms47-CEN; pRS416-Z_3_EV-hphMX-pZ_3_-bL35m-CEN	This study	N/A
COX5a-GFP; pRS416-Z_3_EV-LeuMX-pZ_3_-VN-CEN; pRS416-Z_3_EV-KanMX-pZ_3_-VN-CEN; pRS416-Z_3_EV- hphMX-pZ_3_-VN-CEN	This study	N/A
COX5a-GFP; pRS416-Z_3_EV-LeuMX-pZ_3_-mS46-CEN; pRS416-Z_3_EV-KanMX-pZ_3_-ms47-CEN; pRS416-Z_3_EV-hphMX-pZ_3_-bL35m-CEN	This study	N/A
TOM22-GFP; pRS416-Z_3_EV-LeuMX-pZ_3_-VN-CEN; pRS416-Z_3_EV-KanMX-pZ_3_-VN-CEN; pRS416-Z_3_EV- hphMX-pZ_3_-VN-CEN	This study	N/A
TOM22-GFP; pRS416-Z_3_EV-LeuMX-pZ_3_-mS46-CEN; pRS416-Z_3_EV-KanMX-pZ_3_-ms47-CEN; pRS416-Z_3_EV-hphMX-pZ_3_-bL35m-CEN	This study	N/A
ATP12-GFP; pRS416-Z_3_EV-LeuMX-pZ_3_-VN-CEN; pRS416-Z_3_EV-KanMX-pZ_3_-VN-CEN; pRS416-Z_3_EV- hphMX-PZ_3_-VN-CEN	This study	N/A
ATP12-GFP; pRS416-Z_3_EV-LeuMX-pZ_3_-mS46-CEN; pRS416-Z_3_EV-KanMX-pZ_3_-ms47-CEN; pRS416-Z_3_EV-hphMX-pZ_3_-bL35m-CEN	This study	N/A
*Δtpk3*:KanMX; pRS416-Z_3_EV-LeuMX-pZ_3_-VN-CEN; pRS416-Z_3_EV-HisMX-pZ_3_-VN-CEN; pRS416-Z_3_EV- hphMX-pZ_3_-VN-CEN	This study	N/A
*Δtpk3*:KanMX; pRS416-Z_3_EV-LeuMX-pZ_3_-mS46-CEN; pRS416-Z_3_EV-HisMX-pZ_3_-ms47-CEN; pRS416-Z_3_EV- hphMX-pZ_3_-bL35m-CEN	This study	N/A
Δ*tpk1*:KanMX; pRS416-Z_3_EV-LeuMX-pZ_3_-VN-CEN; pRS416-Z_3_EV-HisMX-pZ_3_-VN-CEN; pRS416-Z_3_EV- hphMX-pZ_3_-VN-CEN	This study	N/A
Δ*tpk1*:KanMX; pRS416-Z_3_EV-LeuMX-pZ_3_-mS46-CEN; pRS416-Z_3_EV-HisMX-pZ_3_-ms47-CEN; pRS416-Z_3_EV- hphMX-pZ_3_-bL35m-CEN	This study	N/A
Δ*mig1*:KanMX; pRS416-Z_3_EV-LeuMX-pZ_3_-VN-CEN; pRS416-Z_3_EV-HisMX-pZ_3_-VN-CEN; pRS416-Z_3_EV- hphMX-pZ_3_-VN-CEN	This study	N/A
Δ*mig1*:KanMX; pRS416-Z_3_EV-LeuMX-pZ_3_-mS46-CEN; pRS416-Z_3_EV-HisMX-pZ_3_-ms47-CEN; pRS416-Z_3_EV- hphMX-pZ_3_-bL35m-CEN	This study	N/A
*Δmig2*:KanMX; pRS416-Z_3_EV-LeuMX-pZ_3_-VN-CEN; pRS416-Z_3_EV-HisMX-pZ_3_-VN-CEN; pRS416-Z_3_EV- hphMX-pZ_3_-VN-CEN	This study	N/A
*Δmig2*:KanMX; pRS416-Z_3_EV-LeuMX-pZ_3_-mS46-CEN; pRS416-Z_3_EV-HisMX-pZ_3_-ms47-CEN; pRS416-Z_3_EV- hphMX-pZ_3_-bL35m-CEN	This study	N/A
*Δtor1*:KanMX; pRS416-Z_3_EV-LeuMX-pZ_3_-VN-CEN; pRS416-Z_3_EV-HisMX-pZ_3_-VN-CEN; pRS416-Z_3_EV- hphMX-pZ_3_-VN-CEN	This study	N/A
*Δtor1*:KanMX; pRS416-Z_3_EV-LeuMX-pZ_3_-mS46-CEN; pRS416-Z_3_EV-HisMX-pZ_3_-ms47-CEN; pRS416-Z_3_EV- hphMX-pZ_3_-bL35m-CEN	This study	N/A
rpl25-6xHis-KanMX; *trp*:: pGDP-preSU9-GFP-APEX2-NatMX	This study	N/A
GFP-tagged mitoribosome proteins	Yeast GFP collection^49^	N/A

Oligonucleotides		

GFP_swap_F2: GGTCGACGGATCCCCGGGTTAATTA	This study	N/A
GFP_swap_R1: GAATTCGAGCTCGTTTAAAC	This study	N/A

Recombinant DNA		

pGAP-PreSU9-mCherry-3xGFP	This study	N/A
β11-NatMX		
pFA6a-GFP_1-10_-3XFLAG-URA	This study	N/A
pRS416-Z_3_EV-LeuMX-pZ_3_-mS46	This study	N/A
pRS416-Z_3_EV-His3MX-pZ_3_-mS47	This study	N/A
pRS416-Z_3_EV-hphMX-pZ_3_-bL35m	This study	N/A
pRS416-Z_3_EV-LeuMX-pZ_3_-mS46-CEN	This study	N/A
pRS416-Z_3_EV-His_3_MX-pZ_3_-mS47-CEN	This study	N/A
pRS416-Z_3_EV-hphMX-pZ_3_-bL35m-CEN	This study	N/A
pRS416-Z_3_EV-KanMX-pZ_3_-mS47-CEN	This study	N/A
pRS416-Z_3_EV-LeuMX-pZ_3_-mS46-yeGFP	This study	N/A
pRS416-Z_3_EV-His3MX-pZ_3_-mS47-yeGFP	This study	N/A
pRS416-Z_3_EV-hphMX-pZ_3_-bL35m-yeGFP	This study	N/A
pRS416-Z_3_EV-KanMX-pZ_3_-mS47	This study	N/A
pRS416-Z_3_EV-hphMX-pZ_3_-VN-CEN	This study	N/A
pRS416-Z_3_EV-KanMX-pZ_3_-VN-CEN	This study	N/A
pRS416-Z_3_EV-LeuMX-pZ_3_-VN-CEN	This study	N/A
pRS416-Z_3_EV-HisMX-pZ_3_-VN-CEN	This study	N/A

Software and algorithms		

Fiji	Schindelin et al.^50^	RRID: SCR_002285
ImageJ	Schneider et al.^51^	RRID: SCR_003070
PyMOL	The PyMOL Molecular Graphics System	Version 2.0 Schrödinger, LLC
UCSF ChimeraX	Meng et al.^52^	RRID: SCR_015872
NIS-Elements	Nikon Instruments Inc	Version 5.42.06

## Highlights

- Metabolic state remodels mitoribosome structure and composition
- Remodeling tunes mitoribosome translation activity to match metabolic demands
- Mitoribosome activity signals to nucleus to regulate mitochondrial biogenesis
- Environmental cues reshape antibiotic sensitivity independently of genetic mutations
