# Supplementary material for: Metabolic environment-driven remodeling of mitochondrial ribosomes regulates translation and biogenesis
Source: Mol Cell. Author manuscript; Available in PMC 2025 Dec 22. (PMC12721022; doi:10.1016/j.molcel.2025.10.012)
Supplement: 5 [file NIHMS2120915-supplement-5.pdf]

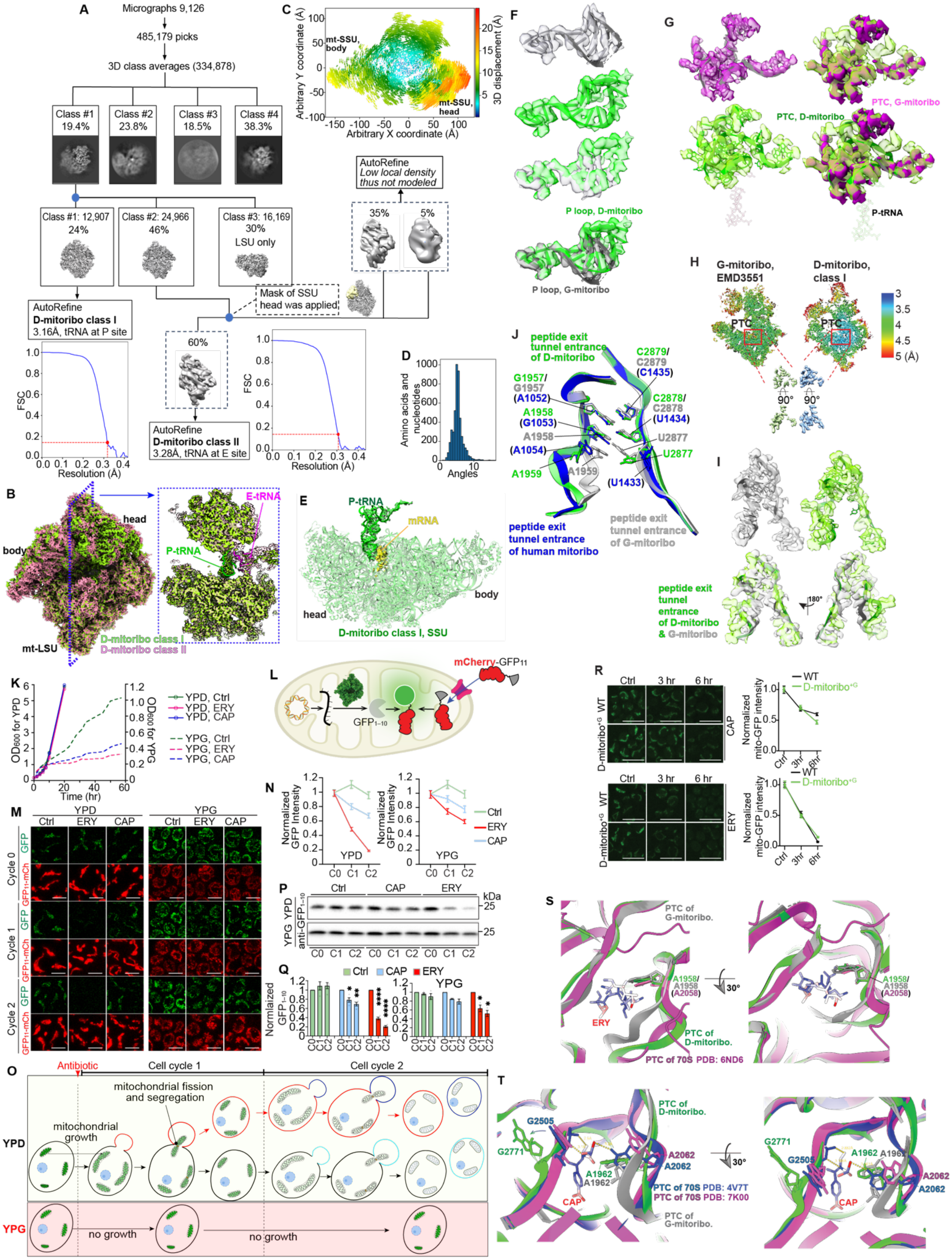

Figure S1. Cryo-EM pipeline and metabolism-dependent remodeling of the mitoribosome, related to Figure 1.

(A) Electron microscopy data processing workflow.
(B-D) The comparison of the mitoribosome structure between D-mitoribosome class I and II. Overall map comparison of D-mitoribosome class I (green) and II (pink), including a cross-sectional view, highlights the differences in tRNA density (B). Quantified distance (C) and angle (D) of atoms displacement between mtSSUs of the two classes of D-mitoribosome after aligning their mtLSU.
(E) The density map of P-tRNA and mRNA in the class I D-mitoribosome.
(F) The density maps of P-loop in D- and G-mitoribosome.
(G) The density maps of PTC from D- and G-mitoribosome.
(H) Local resolution of G- and D-mitoribosome maps. The highlighted regions of the PTC domain (nucleotides 1958–1965, 2713–2720, and 2765–2773) are magnified to illustrate the corresponding map density.
(I) The density maps of peptide exit tunnel entrance from D- and G-mitoribosome.
(J) Comparison of peptide exit tunnel entrance in D-mitoribosome (green) and G-mitoribosome (grey) and human mitoribosome (blue, from PDB ID: 6GAW). Human mitoribosome show high similarity with D-mitoribosome in the expanded entrance, but not with G-mitoribosome.
(K) Representative growth curves of yeast cells cultured under different conditions. CAP and ERY were dissolved into the indicated medium directly. Ctrl, control cells. Same for other figures.
(L) Schematic illustration of the BiG Mito-Split-GFP complementation in yeast.
(M-N) Representative images (M) and quantification (N) of the cell cycle-dependent BiG Mito-Split-GFP changes in YPD and YPG (cell number for each strain refer to Table S2). As YPG-cultured cells did not grow in the presence of antibiotics, we parallelly collected YPG samples at the same time as the YPD samples to ensure the cells were treated with antibiotics for the same amount of time. Intensity of each sample is normalized to the sample before antibiotics treatment (N). Ctrl, control cells without mitoribosome inhibitors. Scale bar: 5 μm.
(O) Schematic illustration of the BiG Mito-Split-GFP signal change upon the addition of antibiotics in YPD and YPG. Ctrl, control cells.
(P-Q) Western blot (P) and quantification (Q) of BiG Mito-Split-$GFP_{1\text{–}10}$ from the samples in Figure S1M. C0, C1, C2: cell cycle 0–2 as in (M). Ctrl, control cells.
(R) Increased expression and incorporation of these $MRPs^{G}$ in D-mitoribosome (D-$mitoribosome^{+G}$) enhanced its sensitivity to CAP compared to the wild-type D-mitoribosome, suggesting that the incorporation of these $MRPs^{G}$ converts the PTC regions—where CAP is known to interact—into a conformation resembling that of G-mitoribosome. Ctrl, control cells.
(S) Comparison of erythromycin (ERY) in both the D- and G-mitoribosome mtLSU structures using bacterial ribosomes bound to ERY as references (PDB: 6ND6).
(T) Comparison of chloramphenicol (CAP) in both the D- and G-mitoribosome mtLSU structures using bacterial ribosomes bound to CAP as references (PDB: 4V7T).

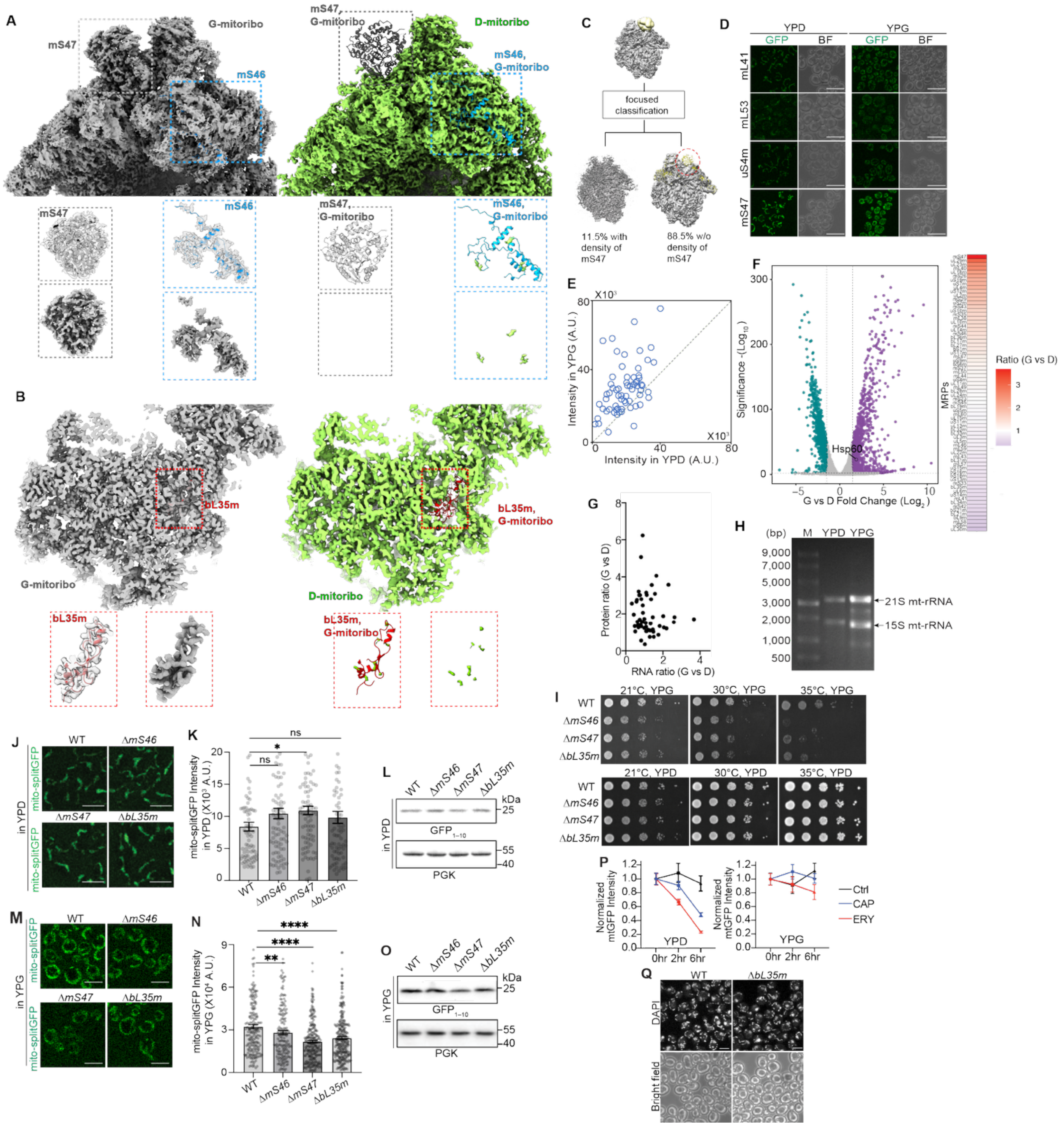

Figure S2. Additional data for the structural and activity difference between D- and G-mitoribosome, related to Figure 2.

(A-B) The density maps of mS47 (gray) and mS46 (light blue) in mtSSU (A) and bL35m (red) in mtLSU (B) of D- and G-mitoribosome. The boxed areas indicate the positions of each protein in the mitoribosomes. For each protein, both the map and the map overlay with the model are shown. For D-mitoribosome, the model of each protein from G-mitoribosome was used to extract the local density from the D-mitoribosome map. As bL35m is located deep inside the mtLSU, a thin section of the mtLSU was shown to illustrate the (lack of) density of bL35m. Note that these three proteins exhibit good density in the G-mitoribosome map but lack density in the D-mitoribosome map.

(C) Focused classification based on MRP mask revealed sub-classes D-mitoribosome with or without mS47. Percentage of 74S D-mitoribosome particles with or without mS47 is shown. G-mitoribosome model (yellow) was fitted to the map to indicate the position of mS47 in D-mitoribosome sub-class lacking mS47 (highlighted in red dashed circle).

(D) Representative images of MRPx-GFP in YPD and YPG. Images are representative of at least two independent

experiments. Scale bar: 10 μm. BF, bright field.

(E) MRPx-GFP abundance in YPD and YPG measured by GFP intensity in each cell.

(F) RNA-seq analysis of gene expression changes in cells cultured in glycerol versus glucose (G vs D). The expression changes of MRPs are shown as heatmap.

(G) Comparison of protein abundance and mRNA expression changes for MRPs in cells cultured in glycerol versus glucose (G vs D) reveals a limited correlation between transcript and protein levels. This suggests that post-transcriptional regulatory mechanisms contribute to the differential expression of MRPs under distinct metabolic conditions. For example, Hsp60 levels are also elevated in glycerol-grown cells (F), indicating enhanced mitochondrial protein folding and maturation in this environment.

(H) mt-rRNA abundance in YPD and YPG. Mitochondria from the same number of cells were used to purify mt-rRNA. M, marker.

(I) Growth assay of G-mitoribosome-specific MRP knockout mutants on YPD or YPG plates. Note that the MRP knock out mutants displayed a significant lower growth rate on YPG plate, while no difference was observed on YPD plate. WT, wild-type cells.

(J-L) Representative images, quantification, and immunoblot of BiG Mito-Split-GFP in different strains cultured in YPD. Scale bar: 5 μm. WT, wild-type cells.

(M-O) Representative images, quantification, and immunoblot of the BiG Mito-Split-GFP signal in G-mitoribosome-specific MRP knockout mutants grown in YPG. The BiG Mito-Split-GFP intensity for each mutant was normalized to that of WT cells. Scale bar: 5 μm. WT, wild-type cells.

Bar graphs are Mean and SEM (Cell number for each strain refer to Table S2).

(P) Quantification of the $sfGFP^m$ (mtDNA-encoded GFP reporters in the W303 background) changes under chloramphenicol (CAP) and erythromycin (ERY) treatment in YPD and YPG (cell number for each strain refer to Table S2).

(Q) Representative images of DAPI staining in WT and Δ*bL35m* cells. Scale bar: 5 μm.

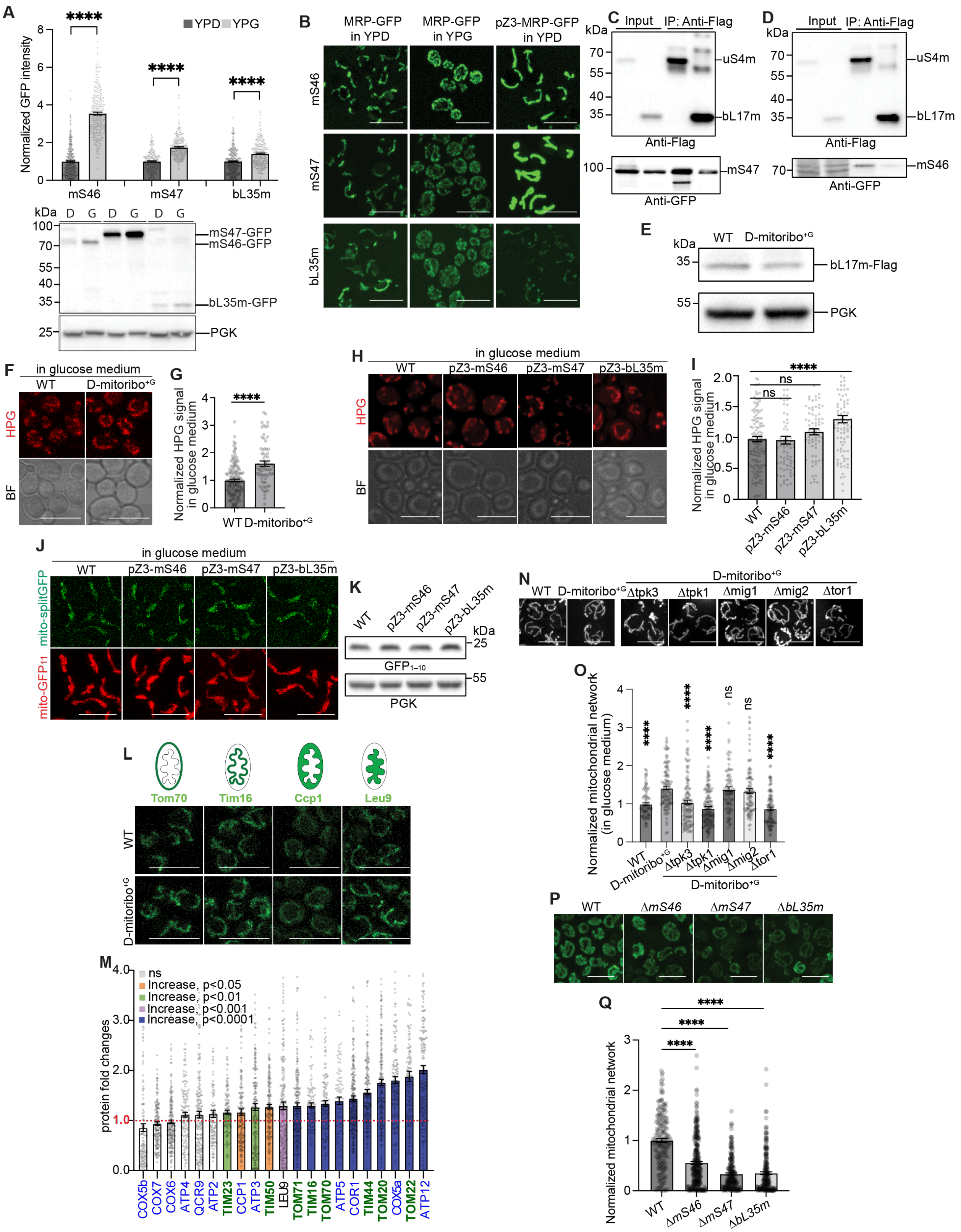

Figure S3. G-mitoribosome–specific MRPs modulate mitochondrial translation and biogenesis, related to Figure 3. **(A)** Quantification of the G-mitoribosome-specific MRP-GFP in YPD and YPG from imaging (top panel) and western blot (bottom panel).

(B) Representative images of the G-mitoribosome-specific MRP-GFP cells grown in YPD and YPG, along with cells exhibiting ectopic leakage expression of G-mitoribosome-specific MRP-GFP driven by the Z3EV promoter in the absence of inducer beta-estradiol.

(C-D) Co-immunoprecipitation of mS47 and mS46 from the leakage expression of Z3EV promoter in (B) with uS4m (mtSSU) and bL17m (mtLSU). Note that both mS47-GFP and mS46-GFP from the leakage expression of Z3EV promoter was pulled down by uS4m-Flag or bL17m-Flag. The co-immunoprecipitation assay for bL35m was not included due to the inability to detect it via western blot.

(E) Western blot of bL17m-Flag in wild-type cells and D-mitoribosome^{+G} cells co-expressing three MRPs^{G} from the leakage expression of Z3EV promoter. Note there is no difference in the expression level of bL17m in the D-mitoribosome^{+G} cells compared to WT. WT, wild-type cells.

(F-G) Representative images and quantification of HPG signal from wild-type cells and D-mitoribosome^{+G} cells co-expressing three MRPs^{G} from the leakage expression of Z3EV promoter, both cultured in glucose medium. WT, wild-type cells.

(H-I) Representative images and quantification of HPG signal from wild-type cells and cells expressing each individual G-mitoribosome-specific MRP cultured in YPD. Each G-mitoribosome-specific MRP was expressed from Z3EV promoter without inducer to achieve its leak expression level. 'pZ3-' here represents leakage expression of the indicated MRP from Z3EV promoter, same in other figures. WT, wild-type cells.

(J-K) Representative images and immunoblot of the BiG Mito-Split-GFP changes in wild-type cells and cells expressing each individual G-mitoribosome-specific MRPs cultured in glucose medium. WT, wild-type cells.

(L-M) Representative images and quantifications of the mitochondrial proteins localized to different sub-compartments of mitochondria in different cells. Mitochondrial proteins were visualized by endogenous C-terminal GFP tagging and expressed from their native promoters. OXPHOS components and TOM-TIM complex are colored by blue and green texts in (M), respectively. WT, wild-type cells.

(N-O) Representative images and quantifications of the mitochondrial network size in different mutants expressing D-mitoribosome^{+G}. The mitochondrial network was stained by MitoTracker™ Red CMXRos. Note that Δtpk1/3 and Δtor1 block the mitochondrial size increase induced by D-mitoribosome^{+G}. WT, wild-type cells.

(P-Q) Representative images and quantifications of the mitochondrial network size in different mutants cultured in glycerol. WT, wild-type cells. The mitochondrial network was stained by MitoTracker™ Green FM.

Bar graphs are Mean and SEM (cell number for each strain refer to Table S2). Data were analyzed with unpaired two-tailed t test. Scale bar: 10 μm.

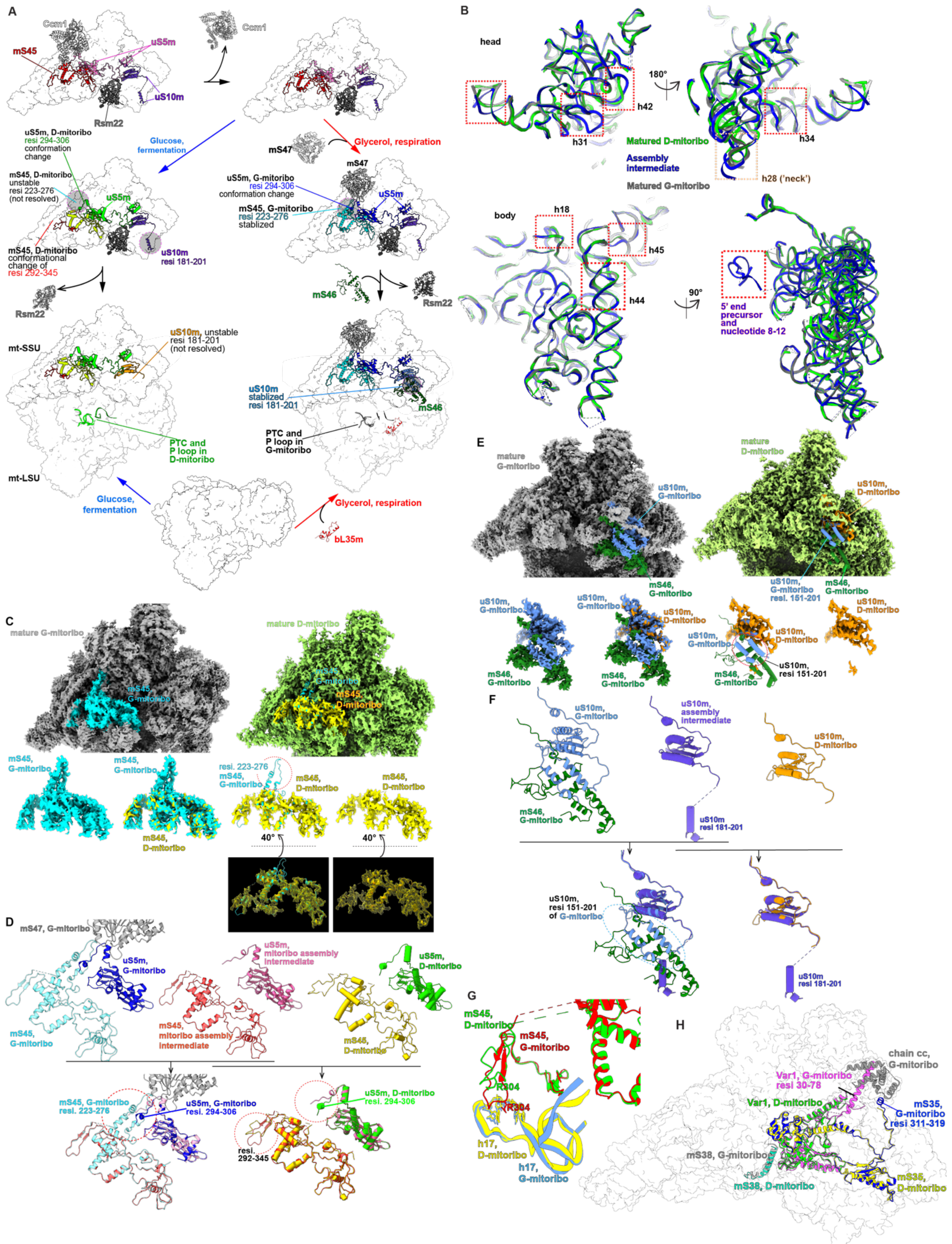

Figure S4. Metabolism-modulated mitoribosome assembly alters MRP conformations, related to Figure 4A-D.

(A) In glycerol medium, the maturation of the mtSSU in the respiring yeast cells is associated with the replacement of Ccm1 by mS47 which changes the conformation of the residue 294–306 of uS5m and stabilizes the residue 223–276 of mS45[46]. uS5m spans across the body-head domain of mtSSU to connect mS47/mS45 with uS10/mS46. These changes in Ccm1-mS47, mS45, and uS5m are followed by the removal of Rsm22 and insertion of mS46, which in term stabilizes the C-terminal 181–201 residues of uS10m[46]. The maturation of mtLSU in the respiring yeast cells is associated with the insertion of bL35m, which is accompanied by the folding change of the PTC and P-loop. In contrast, the maturation of the mtSSU and mtLSU in the fermenting yeast cells take a different path without insertion of mS47, mS46, and the bL35m, leading to the conformation difference of mS45, uS10m, PTC, and P-loop. These mtSSU and mtLSU in the fermenting yeast are functional as they are engaged in active translation with tRNA in their P or E site. The assembly intermediates are from a published paper[46].

(B) In different mitoribosomes, the head and body domains of the 15S rRNA are separated by truncation at h28, which forms the "neck" that connects these two regions. This truncation facilitates the proper alignment of the head and body domains, respectively, even when the swivel statuses differ between mitoribosomes. Regions and helices showing significant structural differences between the assemble intermediate and mature mitoribosomes are highlighted with red dashed line boxes. Notably, D- and G-mitoribosome show similar conformation (overlap) in these helices compared to the immature assemble intermediate. In addition, the mitoribosome assembly intermediate retains the 5' end precursor and nucleotides 8–12 of the 15S rRNA, which are removed during the assembly process and are absent in the mature D- and G-mitoribosome. See Movie S3 for the comparison of 3D structure.

(C) The density map of mS45 in D- and G-mitoribosome. Note that residue 223–276 of mS45 lacks density in D-mitoribosome.

(D) Comparison of mS45-uS5m across different mitoribosomes reveals key differences highlighted by red dashed circles. In mature G-mitoribosome, the additional residues 223–276 of mS45 and the altered conformation of residues 294–306 of uS5m distinguish it from the mitoribosome assembly intermediate (PDB ID: 8D8K). In contrast, mature D-mitoribosome differs from the mitoribosome assembly intermediate in the conformation of residues 292–345 of mS45 and residues 294–306 of uS5m.

(E) The density map of mS46 in D- and G-mitoribosome. Notably, in addition to the missing density of mS46, residues 151–201 of uS10m also lack density in D-mitoribosome.

(F) Comparison of mS46-uS10m across different mitoribosomes reveals key differences highlighted by red dashed circles. In mature G-mitoribosome, the presence of mS46 and the altered conformation of residues 151–201 of uS10m distinguish it from the mitoribosome assembly intermediate (PDB ID: 8D8K). In contrast, mature D-mitoribosome differs from the mitoribosome assembly intermediate solely in the conformation of residues 151–201 of uS10m.

(G) C-terminal 292-345 residues of mS45 in D-mitoribosome adopts a different conformation compared to G-mitoribosome, leading to a reduced interaction with the h17 of 15S rRNA.

(H) The lack of chain cc in D-mitoribosome results in the destabilization and unresolved residue 311–319 of mS35 and residue 30–78 of Var1 in D-mitoribosome. Note that mS38 is not changed between D- and G-mitoribosome.

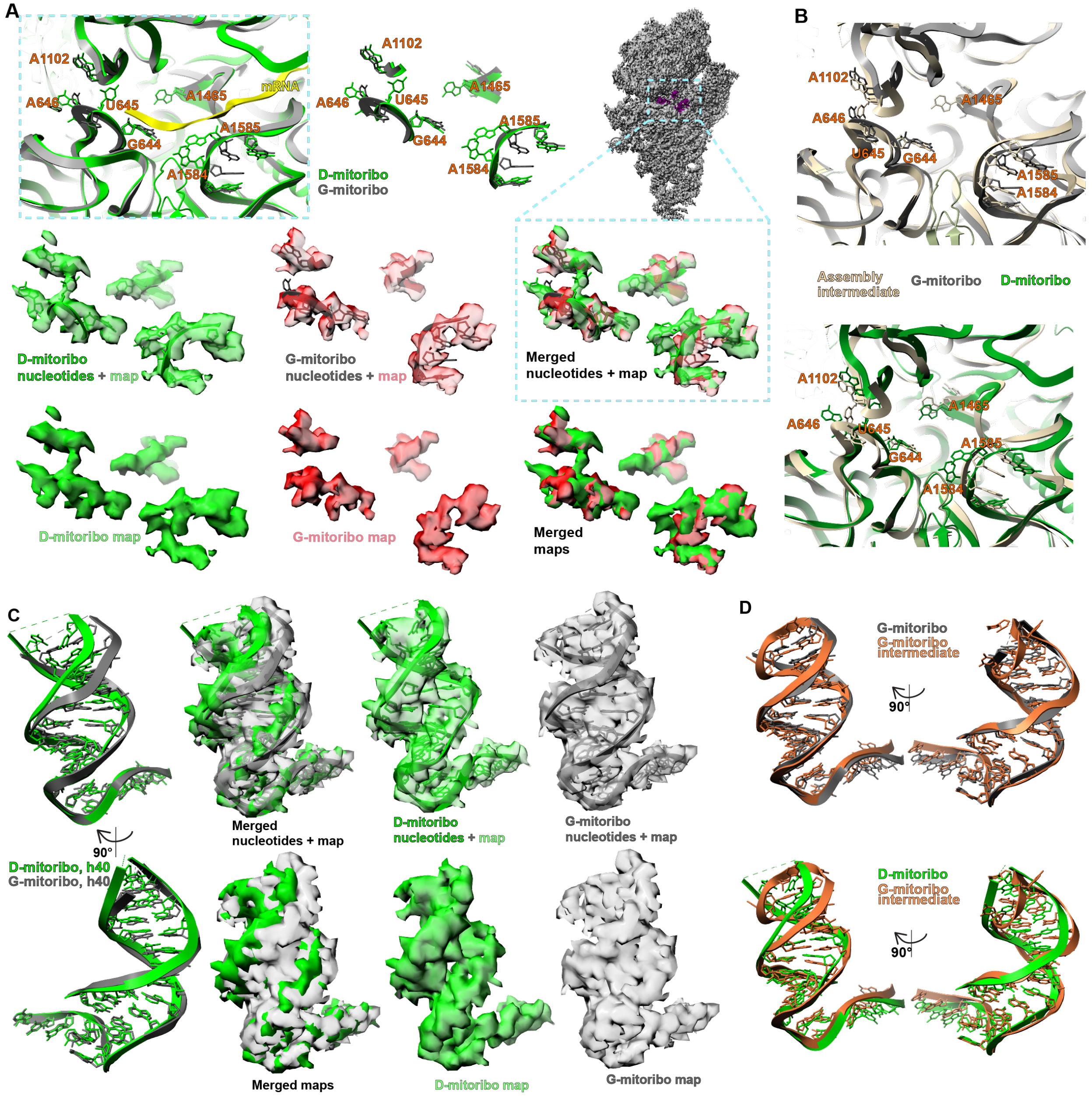

Figure S5. Metabolism-modulated mitoribosome assembly drives conformational shifts in rRNA, related to Figure 4E**.**

(A) The density map of different nucleotides in D- and G-mitoribosome.

(B) Comparison of the conformations of these nucleotides reveals key differences between mature mitoribosomes and assembly intermediates. Notably, the nucleotides that differ in conformation between G-mitoribosome and D-mitoribosome also show differences between the mature mitoribosomes and the assembly intermediate (PDB ID: 8D8K). Therefore, these G- and D-specific conformations likely result from metabolism-specific differential maturation of the mitoribosome for these nucleotides in decoding center.

(C) The density map of nucleotides of h40 in D- and G-mitoribosome.

(D) Comparison of the conformations of these nucleotides reveals key differences between mature mitoribosomes and assembly intermediates. Notably, the nucleotides that differ in conformation between G- and D-mitoribosome also show differences between the mature mitoribosomes and the assembly intermediate (PDB ID: 8D8K). Therefore, these G- and D-specific conformations likely result from metabolism-specific differential maturation of the mitoribosome for these nucleotides in h40.

Supplementary Tables

Table S1: Composition list of D- and G-mitoribosome, related to Figures 1 and 2.

MRPs	Systematic name	D-mitoribosome class I/II MRP chain	G-mitoribosome MRP chain
**21s rRNA**		A	A
**15srRNA**		AL	aa
**tRNA**		AM	bb
**uL2m**	YEL050C	B	B
**uL3m**	YGR220C	C	C
**uL4m**	YML025C	D	D
**uL5m**	YDR237W	E	E
**uL6m**	YHR147C	F	F
**bL9m**	YNR022C	G	G
**uL13m**	YOR150W	H	H
**uL14m**	YKL170W	I	I
**uL15m**	YNL284C	J	J
**uL16m**	YBL038W	K	K
**bL17m**	YJL063C	L	L
**bL19m**	YCR046C	M	M
**bL21m**	YJL096W	N	N
**uL22m**	YNL177C	O	O
**uL23m**	YDR405W	P	P
**uL24m**	YPL173W	Q	Q
**bL27m**	YNL005C	R	R
**bL28m**	YMR193W	S	S
**uL29m**	YLR439W	T	T
**uL30m**	YMR286W	U	U
**bL31m**	YBR122C	V	V
**bL32m**	YCR003W	W	W
**bL33m**	YML009C	X	X
**bL34m**	YDR115W	Y	Y
**bL35m**	YNL122C	N.A	Z
**bL36m**	YPL183W-A	0	0
**mL58**	YKR085C	a	a
**mL59**	YGR076C	b	b
**mL60**	YKL138C	c	c
**mL67**	YDR296W	d	d
**mL38**	YDR322W	1	1

**mL40**	YDR462W	2	2
**mL41**	YBR282W	3	3
**mL43**	YPR100W	4	4
**mL44**	YMR024W	5	5
**mL46**	YNL252C	6	6
**mL49**	YCR071C	7	7
**mL50**	YKR006C	8	8
**mL57**	YLR312W-A	9	9
**bS1m**	YPL118W	g	AA
**uS2m**	YHL004W	h	BB
**uS3m**	Q0140/Var1	i	CC
**uS4m**	YNL137C	j	DD
**uS5m**	YBR251W	k	EE
**bS6m**	YKL003C	l	FF
**uS7m**	YJR113C	m	GG
**uS8m**	YMR158W	n	HH
**uS9m**	YBR146W	o	II
**uS10m**	YDR041W	p	JJ
**uS11m**	YNL306W	q	KK
**uS12m**	YNR036C	r	LL
**uS13m**	YNL081C	s	MM
**uS14m**	YPR166C	t	NN
**uS15m**	YDR337W	u	OO
**bS16m**	YPL013C	v	PP
**uS17m**	YMR188C	w	QQ
**bS18m**	YER050C	x	RR
**uS19m**	YNR037C	y	SS
**bS21m**	YBL090W	z	TT
**mS23**	YIL093C	AG	UU
**mS26**	YOR158W	AH	VV
**mS29**	YGL129C	AI	WW
**mS33**	YGR215W	AJ	XX
**mS35**	YDR175C	AK	YY
**mS37**	YDL045W-A	Z	ZZ
**mS38**	YLR204W	AA	11
**mS41**	YHR059W	AB	22
**mS42**	YJR101W	AC	33

**mS43**	YDR347W	AD	44
**mS44**	YGR084C	AE	55
**mS45**	YGR165W	AF	66
**mS46**	YDR494W	N.A	77
**mS47**	YDR036C	N.A	88
**unknown MRP1**		N.A	cc
**uL10m**	YDL202W	e	N.A
**uL11m**	YNL185C	f	N.A

174
